# Modeling Systems-Level Regulation of Host Immune Responses

**DOI:** 10.1371/journal.pcbi.0030109

**Published:** 2007-06-08

**Authors:** Juilee Thakar, Mylisa Pilione, Girish Kirimanjeswara, Eric T Harvill, Réka Albert

**Affiliations:** 1 Department of Physics, Pennsylvania State University, University Park, Pennsylvania, United States of America; 2 Department of Veterinary and Biomedical Sciences, Pennsylvania State University, University Park, Pennsylvania, United States of America; University of Washington, United States of America

## Abstract

Many pathogens are able to manipulate the signaling pathways responsible for the generation of host immune responses. Here we examine and model a respiratory infection system in which disruption of host immune functions or of bacterial factors changes the dynamics of the infection. We synthesize the network of interactions between host immune components and two closely related bacteria in the genus *Bordetellae.* We incorporate existing experimental information on the timing of immune regulatory events into a discrete dynamic model, and verify the model by comparing the effects of simulated disruptions to the experimental outcome of knockout mutations. Our model indicates that the infection time course of both *Bordetellae* can be separated into three distinct phases based on the most active immune processes. We compare and discuss the effect of the species-specific virulence factors on disrupting the immune response during their infection of naive, antibody-treated, diseased, or convalescent hosts. Our model offers predictions regarding cytokine regulation, key immune components, and clearance of secondary infections; we experimentally validate two of these predictions. This type of modeling provides new insights into the virulence, pathogenesis, and host adaptation of disease-causing microorganisms and allows systems-level analysis that is not always possible using traditional methods.

## Author Summary

The immune response is a complex network of processes activated in a host upon infection. Pathogens seek to disrupt or evade these processes to ensure their own survival and proliferation. This article provides a systems-level analysis of the immune response against two related bacterial species in the *Bordetella* genus, B. bronchiseptica and *B. pertussis. B. pertussis,* the causative agent of whooping cough, has lost many of the virulence factors of its B. bronchiseptica–like progenitor, and is using different strategies for the modulation of the immune system. We have synthesized two separate network models for the interaction of these pathogens with their hosts. Each network is then translated into a predictive dynamic model and is validated by comparison with available experimental data. The model offers predictions regarding cytokine regulation and the effects of perturbations of the immune system, as well as the time course of infections in hosts that had previously encountered the pathogens. We experimentally validate the prediction that convalescent hosts can rapidly clear both pathogens, while antibody transfer cannot substantially reduce the duration of a B. pertussis infection. This type of modeling provides new insights into the virulence, pathogenesis, and host adaptation of disease-causing microorganisms and can be readily extended to other pathogens.

## Introduction

Bacteria persist within their hosts by subverting phagocytosis by immune cells, interfering with antigen processing or presentation [1], or by promoting anti-inflammatory or immunosuppressive responses that normally function to terminate the protective effector immune responses of the host [2]. The dynamic interplay between pathogen and host can have one of three outcomes: death of the host, persistent disease, or recovery. To understand and influence this complex system, it is imperative that we identify the subset of key components and regulatory interactions whose perturbation or tuning leads to significant functional changes. Mathematical modeling can assist in this process by integrating the behavior of multiple components into a comprehensive network model, and by addressing questions that are not yet accessible to experimental analysis.

We used two species of the genus *Bordetellae* as model organisms because (1) they are examples of pathogens that successfully overcome the defenses of their mammalian hosts, (2) their genomes are completely sequenced, and (3) two closely related species of *Bordetellae* provide a comparative model to understand how virulence factors modulate immune responses. The *Bordetellae* are small, Gram-negative coccobacilli, some of which colonize the respiratory tracts of their hosts, adhering to ciliated epithelia and spreading via respiratory droplets. B. bronchiseptica and B. pertussis are two very closely related species that have different host ranges and cause different diseases in their hosts. B. bronchiseptica naturally infects wild and domesticated animals, including leopards, koala bears, cows, dogs, rabbits, and mice [3–5], and causes a persistent disease typified by atrophic rhinitis in pigs and by kennel cough in dogs. *B. pertussis,* which evolved from a B. bronchiseptica–like progenitor, causes whooping cough (pertussis) in humans and is endemic in much of the world. Whooping cough is an acute illness characterized by severe coughing that can become spasmodic, and in some cases leads to death. Although the human pathogen does not cause persistent infection, its rapid spread within relatively dense and mobile human populations is apparently sufficient to allow transient infections to circulate on an ongoing basis, allowing the bacteria to survive within a population.

The different persistence strategies of B. bronchiseptica and B. pertussis are surprising in light of their high genetic relatedness. The *Bordetella* strains mainly evolve through loss of genes and acquisition of insertion sequences. The two strains of *Bordetellae* studied in this paper share 3,394 genes with a synonymous substitution rate of 0.021 [6]. The majority of known virulence factors, including adhesins (filamentous hemagglutinin [FHA], pertactin, and fimbriae) and toxins (adenylate cyclase toxin [ACT] and dermonecrotic toxin) are expressed by both B. bronchiseptica and *B. pertussis.* Despite this, the genome of B. pertussis is 30% smaller than that of *B. bronchiseptica,* due in part to the loss of numerous sizable multigenic regions (e.g., the 22-kb genomic region required for the assembly of a predominant antigen, O-antigen). Interestingly, there also appear to be a number of genes present but not expressed by one pathogen or the other (e.g., the genes encoding pertussis toxin [PTX] are only expressed by *B. pertussis;* see Table 1 [7–12]). Though limited, the genetic variation between B. bronchiseptica and B. pertussis allows substantial differences in their pathogenesis mechanisms.

**Table 1:**
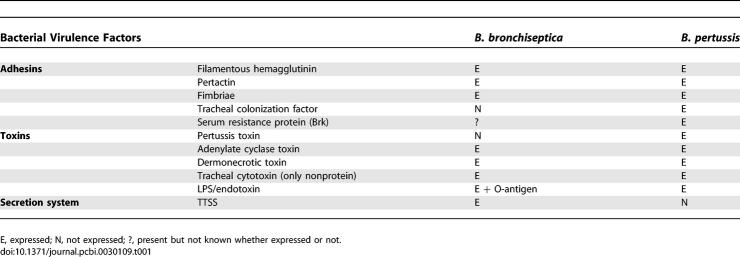
Virulence Factors Expressed by Two Closely Related *Bordetellae*

Mouse infection models with the *Bordetellae* provide an excellent experimental setup in which specific interactions between the host and pathogen can be discovered and manipulated. Both B. bronchiseptica and B. pertussis efficiently colonize the upper and lower respiratory tracts of their hosts and increase in numbers rapidly in the first few days after inoculation. The inflammatory infiltrate, leukocytosis, gradual generation of antibody and T cell responses, and the delayed bacterial clearance from the lower respiratory tract are qualitatively similar to aspects of the clinical pertussis disease. The major aspects of *Bordetellae* virulence and host response have been identified and quantified in the past 20 years, and a wealth of data is available in the literature.

The immune response to a pathogen includes a sequence of processes that are activated by immune cells after sensing bacteria. Here, we construct a network model synthesizing these processes activated in response to the sequenced strains of B. bronchiseptica and *B. pertussis.* We analyze the differences in host immune responses due to the exclusive virulence factors present in the two species by developing separate dynamic models for the infection of the lower respiratory tract by these two species. Discrete dynamic simulation based on the available time course data allows us to monitor the progression of infection in time and to determine the dynamic outcome of *Bordetellae* lung infections. We use the model to predict the outcome of infection scenarios not yet studied and experimentally verify two such predictions.

## Results

### Network Assembly

**Figure 1:**
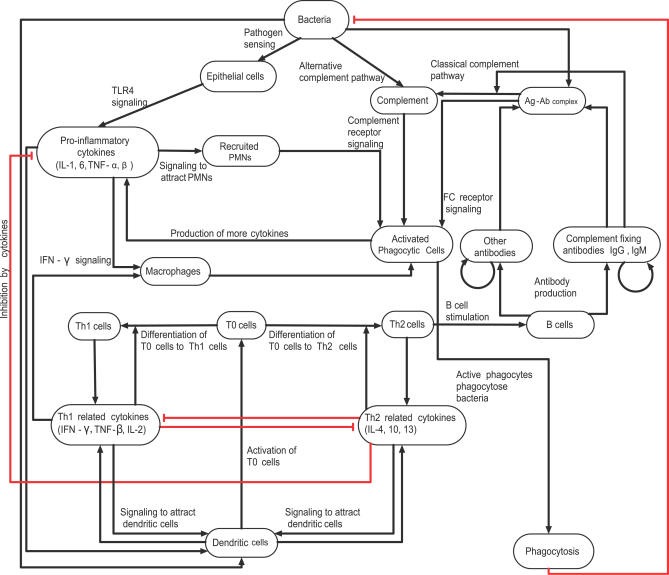
The Consensus Network of Immunological Steps and Processes Activated upon Invasion by *Bordetellae* Species Network nodes denote components of the immune system, and edges represent interactions and processes. Edge labels give a brief biological description of the underlying process. The edges are classified into two regulatory effects, activation and inhibition, and are represented by incoming black arrows and incoming red blunt segments, respectively. Similar notations are used in all network figures.

We started by synthesizing the available data from the literature and from our own experiments (Table S1) into an interaction network (Figure 1). Bacteria and the components of the immune system (i.e., immune cells and cytokines) were represented as network nodes; and interactions, regulatory relationships, and transformations among components were represented as directed edges starting from the source (regulator) node and ending in the target node. We incorporated regulatory relationships that modulate a process (or an unspecified mediator of a process) as edges directed toward another edge. The regulatory effect of each edge was classified into activation or inhibition, and is represented by an incoming black arrow or an incoming red blunt segment, respectively, in the figures. Since not all processes involved in natural *B. pertussis* clearance are known or addressable through the mouse infection model, we extended the set of known interactions by putative interactions based on general immunological knowledge.

We assumed that the bacteria activating the chain of immune responses shown in Figure 1 express the generic adhesins and toxins (Table 1) of the sequenced *Bordetella* strains; we did not assign independent nodes for these virulence factors. However, we did separately include the set of virulence factors identified in the literature as modulating immune responses in a species-specific manner. The resulting network had 18 nodes common in B. bronchiseptica and *B. pertussis;* two species-specific nodes in B. bronchiseptica (O-antigen and the type III secretion system [TTSS]), two species-specific nodes in B. pertussis (PTX, and a combined node for FHA and ACT). Here, we first describe the network common to both species, illustrating the sequence of processes activated after bacterial invasion (Figure 1), followed by a description of the species-specific nodes in the two bacteria.

The first immune mechanisms that respond to Gram-negative bacterial pathogens are Toll-like receptor 4 (TLR4)–mediated recognition of bacteria and the alternative complement pathway. Both species express lipopolyssacharide (LPS) that is recognized by TLR4 receptors on respiratory epithelial cells and dendritic cells (DCs). TLR4-mediated signaling in response to pathogen-associated molecular patterns such as LPS activates the production of cytokines and chemokines. Many of these, including IL-1, IL-6, TNF-α, and TNF-β, are proinflammatory and recruit polymorphonuclear leukocytes (PMNs) to the site of infection [13]. Complement-activated PMNs produce cytokines, which in turn recruit more phagocytes. Following the commencement of phagocytosis, DCs (the main antigen-presenting cells included in the network) present antigens to T0 cells (naive T cells). Signal transduction networks activated during this interaction induce cytokines, leading to the differentiation of T0 cells into either T helper type 1 (Th1) cells or Th2 cells. Th1/Th2 cells also produce the cytokines required for T cell differentiation, leading to a positive feedback in the differentiation process. We denote the common cytokines inducing differentiation of T0 cells to Th1 (or Th2) cells and produced in turn by Th1 (or Th2) cells as Th1 (or Th2)–related cytokines (Th1RCs or Th2RCs). These cytokines are mutually inhibitory in that Th1RCs (such as IFN-γ and IL-12) inhibit the production and function of Th2RCs (for example, IL-4 and IL-10), and vice versa. Some Th2RCs, such as IL-10, are anti-inflammatory, and inhibit the production of proinflammatory cytokines (PICs), reducing the recruitment of PMNs. The balance between the production of Th1RCs and Th2RCs plays an important role in the time course of the immune responses.

Th2 cells activate the clonal expansion of B cells, which in turn produce antibodies against bacterial antigens. Opsonization of bacteria by complement-fixing antibodies leads to the activation of the classical complement pathway. PMNs express complement receptors that recognize complement-coated bacteria as well as Fc receptors that recognize the Fc region of antibodies bound to bacterial antigens; both mechanisms result in the activation of PMNs. In general, the classical complement pathway can directly lyse bacteria; however, this mechanism is found to be weak for *Bordetellae* [14,15]. Therefore, we did not include bacterial lysis in the network. Th1 cells produce a set of cytokines such as IFN-γ, TNF-β, and IL-2, which activate phagocytes to ingest and kill bacteria. PICs and Th1RCs attract more phagocytes to the site of infection, where they are activated and eliminate antibody-bound bacteria. Thus, the Th2- and Th1-mediated adaptive responses constitute a positive feedback to phagocytes for antigen-specific elimination of the pathogen. We assume that the main mechanism of natural clearance of the *Bordetellae* is via phagocytosis by activated phagocytes [14,15].

Bacterial virulence factors such as LPS, PTX, TTSS, FHA, and ACT modulate these host immune processes at several levels. The B. bronchiseptica LPS contains long repeats of O-antigen that inhibit the activation of the alternative complement pathway [16]. B. bronchiseptica expresses a TTSS that induces the necrosis of PMNs [17,18]. The TTSS is known to inhibit the activation of Th1RCs [17]; specifically, the TTSS in association with ACT [19] inhibits IFN-γ production during the first week of the infection, thus inhibiting the differentiation of T0 cells into Th1 cells [20,21]. Consequently, IL-10 (a Th2RC) is produced, facilitating the differentiation of T0 cells into Th2 cells. In a B. pertussis infection, the alternative complement pathway is active [22]; however, early recruitment of PMNs is inhibited by the activity of PTX, and thus PMN activation and phagocytosis are delayed [23]. Like *B. bronchiseptica, B. pertussis* has also developed mechanisms for the suppression of Th1-related responses. FHA [24] and ACT [25], in association with LPS, stimulate IL-10 production by DCs, transiently inhibiting IL-12 and Th1 responses [26]. ACT also enhances DC activation and maturation, promoting the early differentiation of T0 cells into T regulatory 1 (Tr1) and Th2 cells [25,27]. As Tr1 and Th2 cells have a functionally similar role, we represent them as a single node. Note that although the immunomodulation due to the synergistic activity of TTSS and ACT (in B. bronchiseptica) and FHA and ACT (in B. pertussis) appear similar, their exact target of action is different [20,21,27]. Thus, both pathogens have evolved strategies to suppress antigen-specific Th1 responses during the acute phase of infection [28], modulating the balance between Th1 and Th2 responses to their favor.

### Dynamic Model

During the course of infection, the time-dependent expression of specific virulence factors and/or immune components results in a differential infection time course in the two species. We integrated the known temporal information with the interaction network and developed dynamic models for B. bronchiseptica and *B. pertussis* interactions with their hosts. The models incorporate (1) the interactions and regulatory relationships between components (i.e., the interaction network of Figure 1, augmented with the relevant virulence factors), (2) how the strength of the interactions depends on the state of the interacting components (i.e., the transfer functions, given in Table 2), and (3) the initial state of each component in the system. Given the above inputs, the model generates the time evolution of the states of the components of the network (e.g., the time course of bacterial presence, of cytokine concentration, or of immune cell activity).

**Table 2:**
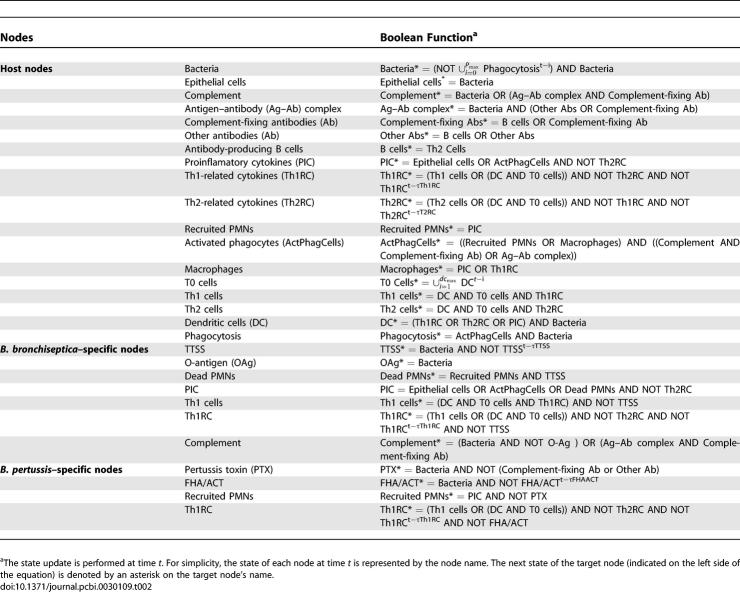
Boolean Functions Used in the Model

Given the scarcity of kinetic and quantitative characterizations of the processes involved in the bacteria–immune system interaction network (Figure 1), we used a discrete dynamic modeling approach. The network's nodes were assumed to have two qualitative states: 0 (off) and 1 (on), corresponding to a baseline (below-threshold) and high (above-threshold) concentration or activity, respectively. The state change of each node was described by a Boolean transfer function *F* that depends on the state of the nodes connected to it by directed edges and on its own state. The transfer functions were developed from the knowledge of the nodes directly upstream of each target node in the network, and augmented with dynamic information from the literature and basic immunology when available. The state of target nodes having a single activator and no inhibitors follows the state of the activator with a delay. Often, the target node is regulated by more than one pathway. We used the AND operator whenever synergy between two (or more) nodes is absolutely necessary to activate the target node. When either of nodes connected to the target node could activate it, the OR operator was used. For inhibition, we used the AND NOT operator, requiring a low level or inactivity for the inhibitor in order for the activation of the target node. Table 2 lists the transfer functions of each node, and a detailed justification of each transfer function is available in Text S1.

The transfer functions define a discrete dynamic system in which iteration determines the evolution of the state of nodes. We employed both the frequently used method of synchronous update [29], using the hypothesis that all regulatory processes have the same duration, and as a more realistic alternative, we used random asynchronous update, where the time scales of each regulatory process are randomly chosen [30–32]. Both methods assume that time is quantized into regular intervals (time steps). The synchronous update can be described as 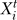 = *F_i_*(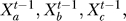 …), where 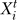 represents the state of node *i* at time step *t*, and *F_i_* is the Boolean function associated with the node *i* and its upstream regulators *a*, *b*, *c*, … . The asynchronous method entails updating the nodes in a randomly selected order during each time step, and interprets the time step as the longest duration required for a node to respond to a change in the state of its regulator(s) (also called a round of update) [30]. In the asynchronous algorithm, the Boolean updating rules are written as 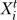 = F_i_(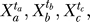 …), where *t_a_*, *t_b_*, and *t_c_* are the time points corresponding to the last change in the state of the input nodes *a*, *b*, and *c* and could be either in a previous or the current time step. Asynchronicity does not change the steady states of the dynamic system, but induces a variability (stochasticity) in the steady states reachable from an initial condition and in the time courses necessary to reach these steady states [31,32].

We extended the general Boolean framework to incorporate known quantitative information by introducing decay rates for cytokines (Th1RCs and Th2RCs) and virulence factors (FHA/ACT and TTSS) and a threshold for the duration of DC activity required for T0 cell differentiation and the duration of phagocytosis necessary for clearance (see Materials and Methods). We performed a systematic search in parameter space to determine the parameter regions that satisfy the following two criteria derived from the literature: (1) reaching bacterial clearance under normal conditions and (2) association of bacterial clearance from the lower respiratory tract with Th1-related activity (see Materials and Methods and Text S2 for a detailed parameter analysis). The two methods of update gave remarkably similar results; thus, in the following we will describe the results obtained by the asynchronous algorithm and refer to the synchronous algorithm only to describe divergent behaviors.

### Relative Duration of Regulatory Immune Processes

**Figure 2:**
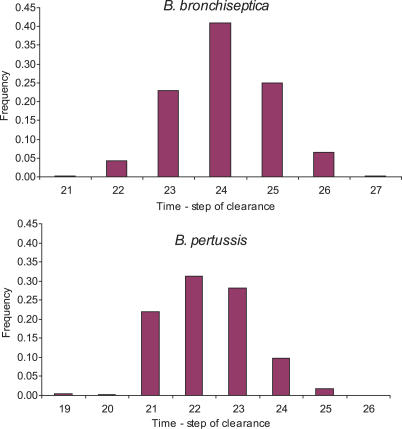
The Distribution of B. bronchiseptica and B. pertussis Clearance Time Steps in the Completely Asynchronous Model The frequency (incidence) of bacterial clearance is plotted as a function of the time step of clearance.

We started with a completely asynchronous algorithm and studied the outcome by monitoring bacterial clearance and the order of activation of immune processes. We performed 1,000 runs with different orders of update selected randomly in each time step. The disease was allowed to evolve for 70 time steps in each simulation. We obtained a distribution of the time steps necessary for bacterial clearance with mean μ = 24 and standard deviation σ = 0.97 in *B. bronchiseptica,* and mean μ = 22.5 and standard deviation σ = 1.2 in B. pertussis (Figure 2).

Next, we aimed to incorporate existing knowledge on the relative duration of the processes represented by single edges in the network. These processes include ligand–receptor binding, signal transduction, cytokine production, cell differentiation, and cellular chemotaxis, and span a range of time scales. We incorporated inequalities between two process durations as updating one node before the other in each round of update. Candidate inequalities were accepted only if the resulting dynamics of the model did not contradict experimentally known temporal information (e.g., that B. bronchiseptica and B. pertussis infections of mice with an initial dose of ~5 × 10^5^ colony-forming units [CFUs] are reproducibly cleared from the lung by days 70 and 50, respectively; see Figure S1 and Tables S2 and S3 for a compilation of known temporal information). First, to incorporate the fact that the residential epithelial cells are first to recognize bacteria, epithelial cells were always updated before DCs in both species. The implementation of this criterion decreased the standard deviation of the clearance time distribution to 0.88 in B. bronchiseptica (μ = 24) and 1.07 in B. pertussis (μ = 22) (see Figure S2A).

Second, because cytokine regulation is important in directing the course of infection and because only limited information was available on the timing of cytokine production and decay, we explored all possible relative durations for the synthesis of the three cytokine classes (PICs, Th1RCs, and Th2RCs), and found that updating Th1RCs before Th2RCs and Th2RCs before PICs led to a significant narrowing of the clearance time distributions (μ = 27, σ = 0.62 in B. bronchiseptica and μ = 22, σ = 0.64 in *B. pertussis;* see Figure S2B). The first part of this condition implies that either the Th1RCs are produced faster than the Th2RCs or Th1RCs are sensed faster than Th2RCs during the interaction between T0 cells and DCs. The validity of this assumption is supported by the experimental observation that in the absence of the immunomodulating virulence factors TTSS/ACT and FHA/ACT, IFN-γ (a Th1RC) is produced before IL-10 (a Th2RC) [20,21,27]. The condition on PICs was necessary only for their second, adaptive immunity–related activation, and implies that the inhibition of proinflammatory cytokines by Th2-related cytokines is released later than their inhibition of Th1RCs (Th1-related cytokines) [22]. The cytokine timing criterion described above was also necessary to reproduce the known earlier clearance of certain mutant bacteria (described in Table S3; see Systemic Effects of Deletions and Comparison with Experimental Results for more information).

Third, to incorporate the fact that the activation of the recruited phagocytes and consequent phagocytosis of bacteria are complex multistep processes, the corresponding nodes were updated penultimately and last, respectively, in each step. After the implementation of these conditions, B. bronchiseptica and B. pertussis were cleared on a single time step, the 26th and 21st, respectively, in all 1,000 simulations. Thus, the sequence of update orders incorporated in the model offers predictions on the population level differences in the relative time scale of the immune processes. Experimental testing of the timing inequalities expressed by our update criteria can potentially elucidate the possibilities for variation in the clearance time scale of natural infections. The next step was to analyze the activity of each node during our in silico pathogenesis.

### Three Phases in the In Silico Pathogenesis

**Figure 3:**
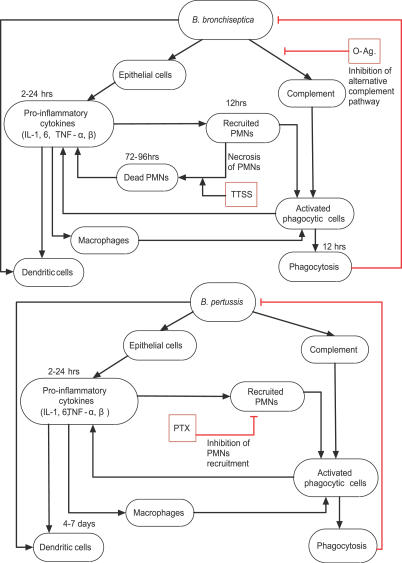
Phase I of In Silico Pathogenesis Depicts innate immune responses leading to activation of DCs, phagocytes, and PICs during the first week of the infection by B. bronchiseptica or *B. pertussis.* Bacterial virulence factors are shown as red squares, and edges corresponding to inhibition are in red.

**Figure 4:**
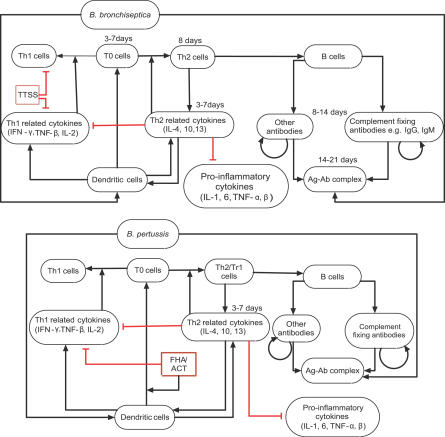
Phase II of In Silico Pathogenesis Depicts Th2-related responses, including antibody production and humoral responses to the infection by B. bronchiseptica or B. pertussis infections. Bacterial virulence factors are shown as red squares, and inhibition is denoted by red edges.

**Figure 5:**
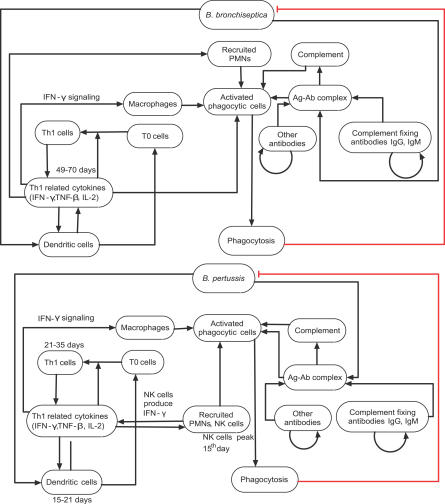
Phase III of In Silico Pathogenesis Depicts Th1-related responses to the infection by B. bronchiseptica or *B. pertussis.*

**Figure 6:**
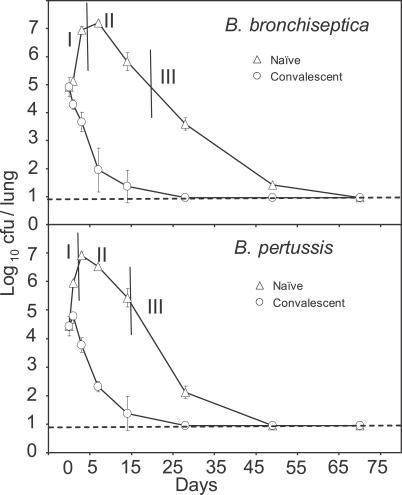
Growth Curves of the Bacteria in Naive and Convalescent Mice Indicating Three Phases of Pathogenesis Open triangles represent the experimentally observed mean ± standard error of bacterial number time courses of B. bronchiseptica (top) and B. pertussis (bottom) infection in naive mice. Vertical lines delineate the three pathogenesis phases, which we identify as phase I: innate immune responses (0–7 d); phase II: activation of Th2-related responses and inhibition of Th1-related responses (8–21 d); and phase III: commencement of Th1-related responses and clearance (22–70 d). Open circles represent mean ± standard error of bacterial numbers in a secondary challenge, by the same species, of convalescent mice on day 49 postinoculation with B. bronchiseptica (top) and B. pertussis (bottom). For each case, four mice from each group were killed on the indicated days, and bacterial burden in the lungs was determined. The dotted line denotes the experimental detection limit.

**Figure 7:**
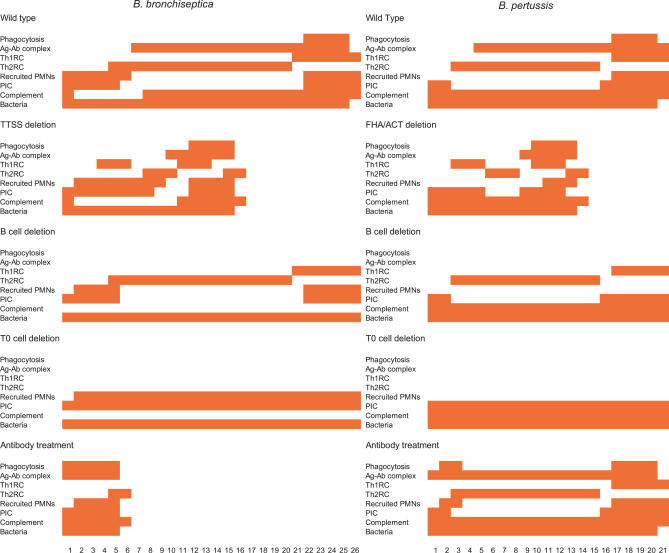
The Pattern of Activation of Key Nodes in the In Silico Pathogenesis Comparison of the time course patterns of key nodes (indicated at the left) in mice infected with B. bronchiseptica or B. pertussis in case of WT, TTSS deletion/FHA/ACT deletion, B cell deletion, T0 cell deletion, and for treatment with antibodies prior to infection. Each colored pattern is a square grid representing the state of the nodes on the *y*-axis versus time steps on the *x*-axis. The colored squares correspond to active nodes (having state 1) at the time step represented on the *x*-axis. One time step of the simulation corresponds to 1 d to 2 d of the real infection.

To represent a previously uninfected host encountering bacteria, we started the dynamic simulation with initial state (at time step *t* = 0) of all nodes at 0 (off), except for bacteria that were assumed to be at 1 (on). We observed three distinct temporal patterns common to all 1,000 simulations with asynchronous update, allowing us to identify three phases in the course of infection: innate responses, including PIC production and the recruitment of PMNs and DCs (phase I); B cell– and antibody-mediated responses (phase II); and Th1-related responses leading to a significant activation of phagocyte recruitment and activation, and ultimately leading to bacterial clearance (phase III). To illustrate these phases of pathogenesis, in Figures 3–5 we present subsets of the network of Figure 1, additionally including species-specific virulence factors; these figures delineate the processes that are most active in the three infection stages within each of the two organisms and indicate timing information whenever an estimate was available (see also Table S4 for a compilation of active nodes in each phase). The experimental data support the existence of distinct responses in these three phases. For example, in the bacterial growth curves shown in Figure 6, the bacterial numbers increase exponentially in the first 7 d of infection (our phase I), after which an immune-mediated decline is observed. While the second and third phases do not clearly separate on the bacterial growth curves, there is ample evidence for the existence of both humoral (Th2-related) adaptive immunity (our phase II) and cellular (Th1-related) adaptive immunity (our phase III) in in vivo infections by wild-type strains. Moreover, both for B. bronchiseptica and *B. pertussis,* recovery from infection was associated with the development of pathogen-specific Th1 cells [33,34]. The long-term steady state of our model after the clearance of bacteria indicates all immune components in an unperturbed (subthreshold) state, with the exception of the two antibody nodes. This steady state is a simplified representation of immunological memory. In the following, we present the in silico time course by focusing on the activity of seven selected nodes (see Figure 7, wild-type [WT]), and we describe the experimental observations supporting our results.

#### B. bronchiseptica.

In phase I, PICs are produced by epithelial cells after bacterial invasion (Figure 7; B. brochiseptica [Bb], WT, PIC). PICs then recruit PMNs to the site of infection (Figure 7; Bb, WT, Recruited PMNs). Indeed, peak cytokine production is observed 2 h after inoculation [13], followed by neutrophil infiltration by 7 h after inoculation in B. bronchiseptica [35,36]*.* TTSS causes necrosis of PMNs [17,18]; however, dying cells induce more PICs, increasing PMN recruitment during the first three time steps. The O-antigen of B. bronchiseptica inhibits the activation of the alternative complement pathway, preventing immediate complement-mediated phagocytosis (Figure 7; Bb, WT, Complement). This phase was five time steps long in the simulation. In phase II of B. bronchiseptica infection, Th1RCs are suppressed due to immunomodulation by the TTSS (Figure 7; Bb, WT, Th1RC), resulting in the production of Th2RCs (Figure 7; Bb, WT, Th2RC). This induces the differentiation of T0 cells to Th2 cells, which facilitates antibody production by B cells (Figure 7; Bb, WT, Ag–Ab complex). Due to the activation of complement-fixing antibodies, complement is active in this phase (Figure 7; Bb, WT, Complement). Th2RCs suppress the production of PICs, inhibiting PMN recruitment (Figure 7; Bb, WT, PIC, Th2RC). Indeed, IL-10 (a Th2RC)–producing cells are observed in B. bronchiseptica–infected mice in the second week after inoculation, due to the inhibition of IFN-γ (a Th1RC)–producing cells by TTSS [20]. The Th1-inhibiting activity of the TTSS decreases below threshold on the 21st time step (see Materials and Methods and Text S2 for more details). This phase lasts 15 time steps in *B. bronchiseptica.* In phase III, the removal of TTSS and Th2RCs results in the production of Th1RCs (Figure 7; Bb, WT, Th1RC; see Figure S1A for the experimental time course of IFN-γ), followed by the activation of phagocytes (Figure 7; Bb, WT, Phagocytosis). In vivo Th1-related immune responses take over around the third week of B. bronchiseptica infection due to the production of IFN-γ [20] (Figure S1). B. bronchiseptica are cleared on the 26th time step (Figure 7; Bb, WT, Bacteria).

#### B. pertussis.

In phase I, PICs are produced by epithelial cells in response to LPS (Figure 7; B. pertussis [Bp], WT, PIC). Unlike in the case of B. bronchiseptica infection, the alternative complement pathway is active (Figure 7; Bp, WT, Complement), but it cannot clear the bacteria (see Text S2). Indeed, TLR4 signaling has been shown to induce PICs in the first 24 h [37], and the alternative complement pathway, although active, was shown to be ineffective in a B. pertussis infection [15]. In the simulation, PTX inhibited the recruitment of PMNs (Figure 7; Bp, WT, Recruited PMNs). Indeed, in mouse models neutrophil infiltration is significantly delayed, and reaches its peak 10 to 14 d after infection [23]. Phase I was three time steps long in the simulation. Phase II starts earlier in B. pertussis than in B. bronchiseptica due to the earlier activation of Th2 cells by FHA/ACT in the model (Figure 7; Bp, WT, Th2RC; refer to Text S2 for the parameter analysis associated with this earlier activation). Indeed, IL-10 is produced through TLR4 signaling in response to ACT and peaks at 24 h after infection [26]. B cells are activated by Th2 cells on the fourth or fifth time step, which is followed by the production of antibodies. The production of Th1RCs is inhibited (Figure 7; Bp, WT, Th1RC) due to the presence of Th2RCs. Antibodies produced in this phase neutralize PTX; however, PMNs are not recruited yet due to the Th2RCs' inhibition of PICs and of Th1RCs. Macrophage activity is also below threshold due to the same inhibition, indicating a lesser pathology of B. pertussis than of B. bronchiseptica in the simulation. In B. pertussis infection, two DC infiltrates have been observed, one in the first week and the other in the second week [38]. We can thus infer that due to the Th1RC inhibition by FHA/ACT, the first DC infiltrate leads to T0 cell differentiation into Th2 cells. Phase III starts on the 15th time step in the simulation. Following Th1RC production, macrophages and PMNs are recruited (Figure 7; Bp, WT, Recruited PMNs) and activated by antibody-opsonized bacteria, leading to B. pertussis clearance on the 21st time step (Figure 7; Bp, WT, Bacteria; see Figure S1B for the experimental time course of PMNs). Phase III also begins earlier in B. pertussis than in B. bronchiseptica because the decay time of FHA/ACT activity, τ*_FHA/ACT_* **,** is shorter than the decay time of the TTSS, τ*_TTSS_* (see Text S2 for a detailed analysis of the decay rates). Indeed, the increased recruitment of natural killer cells stimulates earlier secretion of IFN-γ in B. pertussis infections (explaining the condition τ*_FHA/ACT_* < τ*_TTSS_*), shifting the balance from Th2 responses to Th1 responses in the beginning of the third week (around the 20th day) of the infection [38]. The earlier clearance of B. pertussis is consistent with the notion that B. pertussis infection is rapid and transient and can be sustained in dense populations.

For further validation of the model, we examined the effects of components' deactivation (loss) on the states of other nodes and on the clearance of infection from the lower respiratory tract. The deactivation of components corresponds to loss-of-function mutations: for example, the deactivation of the node TTSS depicts infection by mutant B. bronchiseptica that do not have a functional TTSS. As we discuss below, comparison of such node deletions with the corresponding experimentally studied mutants demonstrates the reliability of the model.

### Systemic Effects of Deletions and Comparison with Experimental Results

We tested the contribution of individual components/nodes on bacterial clearance by simulating their knockout mutants. For each deletion, we set the affected node's state to off (0) and kept it in this state during the simulation. The asynchronous algorithm was run 1,000 times, in each time step generating a new order of update satisfying the three conditions described in Relative Duration of Regulatory Immune Processes. We determined the effect of the node's knockout by monitoring the time step when bacteria are cleared (if at all) and the behavior of other nodes. Figure 7 represents the pattern of active nodes in one representative simulation. The constraints on the relative duration of immune processes led to bacterial clearance during the same time step in all simulated disruptions as well; however, the activation pattern of individual nodes varied slightly depending on the randomly selected order of update.

#### Deletion of TTSS and FHA/ACT.

TTSS in B. bronchiseptica and FHA/ACT in B. pertussis inhibit the production of Th1RCs in phase II (Figure 7; WT, Th1RC). Though the exact mechanism of the inhibition is unknown, it is fairly well established that this inhibition leads to the differentiation of T0 cells to Th2 cells in phase II because of the production of Th2RCs by DCs [19,21]. Deletion of the node TTSS from B. bronchiseptica led to oscillations of Th1RCs and Th2RCs (Figure 7; Bb, TTSS deletion, Th1RC, Th2RC), followed by the activation of antibodies and phagocytes and bacterial clearance on the 16th time step instead of the 26th (Figure 7; Bb, TTSS deletion, Bacteria). This simulation could reproduce the two peaks of Th1RCs, exemplified by IFN-γ, in the experimental time course (compare Figure 7 [Bb, TTSS deletion, Th1RC] with Figure S1A). The early clearance of TTSS-deficient B. bronchiseptica observed in the simulation accurately depicts experimental results with TTSS knockout mutants (see Table S3) [20,21]. The deletion of the node FHA/ACT from B. pertussis led to the clearance of bacteria on the 14th time step instead of the 21st (Figure 7; Bp, FHA/ACT deletion, Bacteria). The combined disruption of FHA and ACT has not yet been performed experimentally, but the model result is consistent with known information on FHA and ACT [27]. Figure S3A shows the distributions of bacterial clearance time steps in case of TTSS or FHA/ACT deletion in the completely asynchronous model (without the timing inequalities described in Relative Duration of Regulatory Immune Processes). The distributions are much broader than the clearance time distributions of the respective WT bacteria (compare with Figure 2), and indicate a considerable chance for the defective mutants being cleared later than WT bacteria, a clearly unrealistic result. Thus, the TTSS and FHA/ACT deletion studies provide additional support for the identified timing inequalities between Th1RCs, Th2RCs, and PICs, and also suggest that in infections by WT *Bordetellae,* the processes activated by Th1 and Th2 cells switch reproducibly due to antigen-specific responses.

#### Deletion of B cells.

The deletion of B cells results in the lack of antibody production. In our model, neither B. bronchiseptica (Figure 7; Bb, B cell deletion) nor B. pertussis (Figure 7; Bp, B cell deletion) was cleared in this disruption, although three distinct infection phases were still observed. B. bronchiseptica showed two peaks of recruited PMNs and PICs, first in phase I and then in phase III (Figure 7; Bb, B cell deletion, Recruited PMNs, PIC). In B. pertussis infection, there was no recruitment of PMNs because the PTX node remained active in the absence of antibodies (Figure 7; Bp, B cell deletion, Recruited PMNs). Complement activation was absent in B. bronchiseptica (Figure 7; Bb, B cell deletion, Complement) in contrast to *B. pertussis,* where the alternative complement pathway was still active (Figure 7; Bp, B cell deletion, Complement). Production of Th1RCs and Th2RCs was unaffected when compared with WT (Figure 7; Bb, Bp, B cell deletion, Th1RC, Th2RC). Note that although Figure 7 stops at the 26th and 21st time steps in B. bronchiseptica and *B. pertussis,* respectively, the observed absence of clearance was independent of the length of the simulation. Experiments show that B. bronchiseptica persists indefinitely in the trachea and lungs of B cell–deficient mice [39]. Also, in the case of *B. pertussis,* although bacterial numbers decrease, no clearance is observed [39], in agreement with our results.

#### Absence of adaptive immunity.

Deletion of T0 cells in the model led to the absence of Th1, Th2, and B cells (Figure 7; Bb, Bp, T0 cell deletion, Th1RC, Th2RC), resulting in bacterial persistence. Although there were no T and B cells, the other immune components responded to the bacteria. During B. pertussis infection, complement was constitutively active due to the active alternative complement pathway (Figure 7; Bp, T0 cell deletion, Complement). PICs were constitutively active in both species (Figure 7; Bb, Bp, T0 cell deletion, PIC), whereas recruitment of PMNs was observed only in B. bronchiseptica infection (Figure 7; Bb, T0 cell deletion, Recruited PMNs). There were no discernible phases for this deletion. The node phagocytosis was off in both species, implying that if there is any phagocytosis, it is under threshold levels. Experiments with defective adaptive immunity in mice indicate that adaptive immunity is indeed required to clear both organisms from the lower respiratory tract [22].

#### Deletion of other immune components.

When DCs were deleted, the response was very similar to the deletion of T0 cells, as DCs are essential for the maturation and differentiation of T0 cells. Note that in our model we have attributed all antigen presentation functions to this node, which therefore represents other potential antigen-presenting cells as well. In the absence of Th2 cells**,** B cells were not activated; hence, the bacteria were not cleared due to the absence of antibodies (similar to Figure 7; Bb, Bp, B cell deletion). Deletion of Th1 cells did not affect clearance because Th1RCs, the only effector elements of Th1 cells, are produced during DC–T0 cell interaction, and these nodes are unaffected by the disruption. Our model cannot incorporate the quantitative increase in Th1RCs produced by Th1 cells; it remains to be seen whether this increase is required. In the absence of macrophages, B. bronchiseptica and *B. pertussis* were cleared on the same time step as in the WT infections. This suggests that phagocytes activated only by Th1RCs are not absolutely required for clearance because the second wave of PICs produced after the decay of Th2RCs is able to activate recruited PMNs. Further quantitative experimental information will be necessary to analyze the relative contribution of Th1RCs and PICs in phagocyte recruitment. Note that Th1 cell deletion and macrophage deletion in the completely asynchronous model (without timing inequalities) broadened the clearance time distribution (compare Figures S3B and S3C with Figure 2); thus, some variability is expected if the timing inequalities are not completely satisfied.

#### Treatment with antibodies prior to infection.

Antibody treatment before or shortly after infection can be used as a prophylaxis. Hence, we simulated its effect by activating the “antibodies” node during the first time step. The earlier treatment with antibodies results in the opsonization of bacteria soon after they invade the host. Antibody treatment led to immediate activation of the node phagocytosis in B. bronchiseptica infection (Figure 7; Bb, Antibody treatment, Phagocytosis). During B. pertussis infection, PMNs are recruited following the PTX's neutralization by antibodies; however, this recruitment is transient (Figure 7; Bp, Antibody treatment, Recruited PMNs) and cannot sustain sufficient phagocytosis (Figure 7; Bp, Antibody treatment, Phagocytosis)*. B. bronchiseptica* was cleared by the sixth time step (Figure 7, Bb, Antibody treatment, Bacteria), but the pre-initiation of antibodies did not lead to early clearance of B. pertussis (Figure 7; Bp, Antibody treatment, Bacteria). In agreement with our model, experimental studies indicate that the adoptive transfer of serum antibodies results in the clearance of B. bronchiseptica by day 3 after inoculation, but B. pertussis is not cleared earlier [39]. Our results are also consistent with human clinical trials, where serum antibody titers could not be correlated with protection against B. pertussis [23,40,41]. The bacterial clearance time steps upon antibody treatment in the completely asynchronous model (Figure S3D) have a broader distribution but agree with the fundamental difference between the time scales of antibody-mediated clearance in B. bronchiseptica and *B. pertussis.*

### Modeling of Secondary Infection

**Figure 8:**
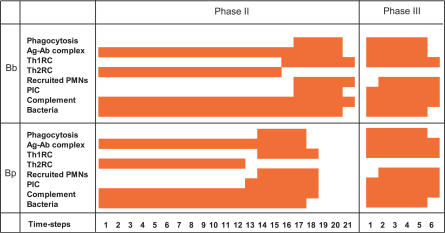
The Pattern of Activation of Key Nodes during Secondary Infections Time course patterns of key nodes (*y*-axis) plotted against time steps (*x*-axis) during secondary infection by B. bronchiseptica (Bb) and B. pertussis (Bp). One time step corresponds to 1 d to 2 d. The initial state corresponds to the activity of immune system components at the start of the secondary infection. Two scenarios are shown: secondary infection given in phase II of the primary infection (third column), or in phase III of the primary infection (fourth column).

To analyze the efficiency of immune responses in detecting and clearing a second challenge with pathogens, we performed simulations of secondary infections. The secondary infection with the same or different species (cross-infections) allowed us to better understand the complex pathogen–host interplay and to generate testable predictions. The secondary infection was modeled by a “secondary” initial state comprising the state of all nodes in the stage of the primary infection when the host encountered the second challenge. This representation of the secondary challenge is not simply a continuation of the primary infection. Although the state of the node “bacteria” does not change at the time of the second challenge (it already is on), the second challenge can lead to the reactivation of certain cytokines and virulence factors that have previously decayed during the primary infection. We modeled three scenarios: first, reinfection of diseased hosts, by using a secondary initial state corresponding to time step 9, in phase II, for both B. bronchiseptica and *B. pertussis.* Second, we modeled the infection of convalescent hosts by a secondary initial state corresponding to time step 21 for B. bronchiseptica and time step 18 for *B. pertussis,* in phase III (refer to Figure 7 for the state of key nodes at these time steps). Third, we modeled the reinfection of hosts with immunological memory by a secondary initial state that has antibodies on. In summary, the “secondary” initial state indicates the state of the host when the second bacterial invasion takes place (see Materials and Methods), and is different from the initial state of the primary infection (where only bacteria are on) and from a continuation of the primary infection. Correspondingly, the in silico pathogenesis of the secondary challenge showed a different pattern of node activation than the time course of a primary infection (compare Figure 8 with Figure 7).

We first studied secondary infections by the same species. We simulated the secondary initial state in phase III by preserving the states of TTSS and FHA/ACT off, assuming that the host recognized those virulence factors of the new bacteria and removed them (or became desensitized). Secondary dosage given in a particular phase generally reset the pathogenesis to the beginning of the same phase of the combined infection, with a few notable exceptions. When the secondary dosage was given in phase II of the primary infection, the antigen–antibody complex was active starting with the first time step of the secondary infection, and phase II of the combined infection was one time step shorter than phase II of the primary infection for both species. When the dosage was given in phase III, the recruited PMN peak was delayed compared with the primary infection in *B. pertussis,* but phagocytosis was immediately activated. We found that the secondary infection was cleared faster than a single (primary) infection in both species and both initial conditions. Secondary infection in phase II (i.e., of diseased hosts) took longer to clear (21 time steps in B. bronchiseptica and 18 time steps in *B. pertussis;* Figure 8, third column), whereas secondary infection in phase III (i.e., of convalescent hosts) was cleared faster (six time steps in B. bronchiseptica and in *B. pertussis;* Figure 8, fourth column). The clearance was always associated with Th1-related responses (phase III). Thus, immune components active in phase III led to early clearance of a secondary infection. In conclusion, the phase of the primary infection when the host encountered a second dose of *Bordetellae* had an important effect on the progression of the second infection**.**

We simulated B. pertussis after B. bronchiseptica and B. bronchiseptica after B. pertussis cross-infections exploring scenarios with or without antibody cross-reactivity (see Text S3 and Table S5). We found that the only case of faster clearance is secondary infection of convalescent hosts in the presence of antibody cross-reactivity, a result confirmed by preliminary experiments (Wolfe DN and ETH, unpublished data).

We also studied secondary infections of immune hosts by using an initial condition where antibodies are active, a state that approximates immunological memory. It is known that memory T cells and B cells are also generated after an infection; however, these cells require reactivation, and the mechanisms involved are not understood. The capacity of memory B cells to proliferate faster after priming and to produce antibodies with increased affinity could not be incorporated in our current qualitative model. Our approximation thus serves as an upper limit of immunological memory. We found similar results as in the case of prior treatment with antibodies (i.e., B. bronchiseptica was cleared by the sixth time step, but there was no early clearance of B. pertussis). This latter result suggests the possibility of reinfection of previously vaccinated individuals, probably associated with a subclinical disease but a nonzero transmission probability [42].

#### Analysis of the effect of active nodes.

To find the minimum number of immune components that must be active at the beginning of a secondary infection by an identical or cross-reacting species to enable early clearance, we turned off the active nodes of the secondary initial state in phase III one by one. We found that only one active immune component, namely one of the two groups of antibodies, is required, since Th1RCs produced early in the secondary infection (due to the absence of TTSS or FHA/ACT) recruited phagocytes that were activated by antibodies and cleared the bacteria. Thus, the minimum condition for the early clearance is that the node “Th1RC” and one of the antibody nodes are on and that TTSS (in B. bronchiseptica) or FHA/ACT (in B. pertussis) is off. This early clearance is based on antigen recognition by the antibodies from the primary infection, similar to earlier clearance in the presence of serum antibodies. Interestingly, our model predicts that the most active phagocytes in secondary infection of convalescent hosts are different from the phagocytes responsible for serum antibody–mediated clearance; namely, the former are macrophages activated by Th1RCs, while the latter are neutrophils recruited by PICs and activated by antibodies. Thus, although macrophages are not required for the clearance of a single infection (see Deletion of other immune components), they play a crucial role in the early clearance of secondary infections of convalescent hosts.

#### Experimental observations validate model predictions.

To test the prediction of earlier clearance of a secondary infection, we performed follow-up experiments where a B. pertussis or *B. bronchiseptica* second challenge was given on day 49 after inoculation. This reinfection of convalescent hosts corresponds to a secondary initial condition of phase III in our simulations. As shown by the open circles in Figure 6, the secondary infections of B. bronchiseptica and B. pertussis were cleared in 15 d. Furthermore, the earlier clearance of the B. pertussis secondary infection, as compared with a B. pertussis time course in the presence of serum antibodies [39], supports the prediction of distinct phagocyte subsets activated by distinct signals. Comparing these results with the model prediction in Figure 8 (fourth column), where bacteria were cleared on the sixth time step, indicate that the model predictions were validated. The predictions for the secondary infection clearance patterns for the remaining combinations will be tested in future studies.

## Discussion

### Network Assembly

Immunological responses have been traditionally studied by biochemical and molecular biology techniques. These approaches allow us to manipulate components that are experimentally detectable, and they increase our knowledge about individual immune mechanisms and related responses. In the present study, we synthesized these separate sets of information into an integrated network (Figure 1) that gives a comprehensive view of the system. Immunological studies often focus on model organisms; however, the results ultimately need to be applicable to the natural host. We overcame such limitations by constructing unbiased (consensus) networks such as that represented in Figure 1 and adding on pathogen-specific mechanisms as specific nodes and edges. Although the edges of the network are based on information from experimental observations, the network is more than the sum of its parts because it enables the evaluation of the direct and indirect effects of perturbing each node. Constructing such consensus models has the potential for accelerating new discoveries in a field; such advances are sorely needed for *B. pertussis,* which is still persistent in human populations in part because the information about human-specific immune responses is limited. Though our network analysis is dependent on the definition of the nodes and edges, it is flexible enough to describe the system under study accurately.

### Dynamic Simulation

The network-based dynamic model enabled us to analyze the time course of the immunological responses and bacterial clearance. Dynamic modeling usually employs continuous or discrete methods. For continuous models, detailed information about the interaction kinetics, rate constants, and component concentrations is necessary [43,44]. Previous models of immunological response to pathogen invasion, mostly based on ordinary differential equations [45,46] or cellular automata models [47], have focused on the interaction between a few cell types, cytokines, and pathogens. The kinetic parameters of these models were estimated by comparison of pathogen and/or cytokine concentration time courses from experiments and models. By focusing on a small subset of the immune response, these models do not reflect the diversity, complexity, and long-range feedbacks present in pathogen–host interactions, and thus may lead to unrealistic results. In our comprehensive network model, several nodes represent populations of cells or families of cytokines, and edges represent whole signal transduction pathways; thus, the molecular-level description of each node would need quantitative knowledge of complex subnetworks, knowledge that is currently missing. The fact that we have limited knowledge, even at the coarse-grained level presented here, does not allow us to use continuous methods.

The qualitative dynamic descriptions we use, in addition to being the practical choice, are well suited for networks that need to function robustly despite changes in external and internal parameters [48,49]. Qualitative discrete modeling such as ours has been previously successfully implemented in gene regulatory networks and signal transduction networks for predicting the dynamic trajectory of biological circuits and for accessing the reliability of gene regulatory networks in signal processing [31,32,50]. In the study of immunological responses, this approach has been implemented in small networks for the analysis of T cell activation and anergy [51] and for the analysis of lymphocyte subsets [52]. Here, a comprehensive network was constructed to study the immunological responses at the systems level, and the dynamic model of this network was successfully validated.

The ingredients (node states, transfer functions) of our dynamic model refer to the node (component) level, and there is no explicit control over pathway-level effects. Moreover, the combinatorial transfer functions we used are, to varying extents, conjectures, informed by the best available experimental information. For example, FcγR, C3, and Th1-mediated activation of phagocytes might have a complex, partly redundant, and partly synergistic relationship, as there is a requirement for both the recruitment and activation (by at least one mechanism) of phagocytes in the natural clearance of both B. bronchiseptica and *B. pertussis.* Our assumption for the node “activated phagocytes” allowed all three processes mentioned above to contribute towards bacterial phagocytosis, and the dynamic model allowed us to analyze the temporal separation between contributions to phagocytosis by these three processes. As the model's dynamics is an emergent, systems-level property, and our choice of parameters was based on the normal time course, an agreement between experimental and theoretical results of node disruptions is not inherent, and provides a validation of the model. Indeed, our model agrees with experimental results on numerous negative or positive perturbations in immune mechanisms (such as the deletion of B cells or adoptive transfer of antibodies).

We incorporated possible stochastic differences within individual host responses by randomly sampling update orders, and incorporated known relative temporal information on interactions and processes as restrictions on the order of update; for example, epithelial cells were always updated before dendritic cells. At a molecular level, we can interpret the restrictions on the order of updates either as restrictions on the relative duration of processes or on the time required to activate their target nodes. For example, updating Th1RCs before Th2RCs either means that the signal transduction pathway activating Th1RCs is faster or that Th1RCs once produced can activate downstream processes more quickly than can Th2RCs. Moreover, the orders of updates also gave an insight into the regulatory processes; for example, we find that a slow phagocytosis process ensures better, more reproducible bacterial clearance. The model identified two possible oscillatory behaviors of Th1RCs and Th2RCs: short oscillations in the absence of modulating factors, and the switch-like behavior in the presence of those modulating factors.

### Virulence Factors

Our network model gives insight into how virulence factors change the course of innate and adaptive immune responses, and allows the comparison of species-specific virulence factors. For example, both the B. bronchiseptica O-antigen and the B. pertussis PTX inhibit early immune responses, assisting bacterial multiplication and survival. Our directed network representation allows for tracing the sequence of immune responses following recognition of the pathogen. The O-antigen interacts with host components earlier in this sequence (in the pseudodynamic sense introduced in [53]) than does PTX, as it inhibits bacterial recognition, while PTX disables the recruitment of immune cells that recognize bacteria. However, the inhibitory effect of PTX is ultimately more effective since it gives B. pertussis a means to resist the effect of serum antibodies. Comparing the way in which the B. bronchiseptica TTSS and the B. pertussis FHA/ACT modulate T helper cell balance gives insight into why the TTSS gene is not expressed in *B. pertussis.* The TTSS causes necrosis of PMNs [17], which is possibly a side effect of the evolution of the TTSS's primary role to modulate cytokines, and which ultimately leads to a stronger immune response. Unlike *B. bronchiseptica,* the milder pathology during B. pertussis infection allows it to modulate immunity silently.

The model also provides insight into regulatory mechanisms of antigens and suggests differential regulation of virulence factors by two distinct mechanisms: (1) immune-mediated neutralization/elimination, or (2) decay due to bacterial gene regulation or to a reduction in bacterial numbers. The conclusion that the effects of the secreted factors ACT and TTSS are longer lasting than the effects of PTX is supported by the observation that PTX does not modulate cytokines or chemokines [23]. The model also predicts that the decay rates of the (effect of) Th2RCs, TTSS, and FHA/ACT are similar. Results of new experiments will lead to further refinement of the model; for example, the speculations that the LPS containing O-antigen decreases host cells' access to other factors such as adhesins, or that it facilitates secretion of toxins, can be incorporated if supported by dynamic evidence.

### Pathogenesis

One of the most important contributions of our model is the identification of the pathogenesis phases. The definition of three infection phases is preferable to using exact timing because specific processes can have different durations in different hosts, and timing results obtained in model organisms might not be directly applicable to the pathogens' natural hosts. The time scale of processes in our dynamic model does not correspond exactly to the experimentally observed timing in mice infections, but the transition from one phase to another is captured. Comparing the model and experimental time scales, we estimate that one time step corresponds to 1–2 d of infection. The time span of the third phase is shorter in the model than in experiments because quantitative differences cannot be reproduced in the present qualitative model; or equivalently, the implicit bacterial concentration threshold of our model is higher than the experimental detection threshold.

### Implications

Models such as this allow the efficient use and logical representation of available information. We extracted host- and pathogen-specific processes from the experimental literature, and used overlapping information, such as the modulation of Th1 and Th2 cell types, from other host–pathogen systems. Our model allows the identification of new relationships and the making of new predictions that would be difficult to derive from less formal analysis. First, the logical network representation of the pathogen- and host-related information will allow microbiologists and immunologists to see the knowledge gaps in the results from different laboratories and appreciate the synergy between the pathogen and the host. For example, the role of FHA in cytokine balance and its possible synergy with TTSS and ACT has not been studied in the ancestral pathogen *B. bronchiseptica.* Second, the timing constraints and parameters derived from our dynamic model give predictions regarding the time scales of several of the processes. Experimental testing of our results on the degradation of toxins and cytokines (or their effects) will be able to establish the mechanism responsible for this degradation. Third, and perhaps most important, the model is able to predict the outcome of perturbations not yet explored experimentally and to direct future experimental efforts. A considerable amount of sustained manual effort is necessary to study immunological processes by traditional techniques. Modeling leads to efficient ways for analyzing the effect of knockout mutations, as it is straightforward to delete certain components in the model and observe the consequences on bacterial clearance and activation/inhibition of immunological components. While our model lacks quantitative kinetic and spatial detail, it can serve as a scaffold to which experimentalists and modelers can add future results on the regulation between immune components and bacterial mechanisms as they become available.

*Bordetella* evolution is leading to the frequent emergence of new strains and species. Our simulations of cross-infections with or without cross-immunity constitute the first step toward modeling the within-host effects of newly emerged *Bordetella* species. Such simulations can be extended to incorporate different combinations of known virulence factors, or to explore putative new virulence factors, providing reasoned expectations prior to costly and time-consuming animal experiments. As population-level dynamics are shaped by within-host interactions [42], models such as ours can increase our understanding of the population-level effects of specific molecular evolution. Infections by two different strains or species can result in a similar outcome (e.g., persistent disease) in terms of bacterial clearance, but have different pathological effects. These species-specific aspects can be readily studied in our model by systematically comparing the simulated dynamics of the immune system in the two species. Comparison between models of different strains and species will allow us to recognize crucial virulence mechanisms and can give us insight into the evolution of new virulence mechanisms.

### Medical Implications

The present study led us to the identification of the three phases in *Bordetella* infections. Putative medical treatments can thus be evaluated in silico by simulating them through adding/removing or activating/inhibiting certain nodes and studying their effect on these three phases. Qualitative conditions (e.g., for cytokine regulation) provided by such models can be used in the future to detect the phase of infection in patients, and ultimately to predict recovery and design medication. Some of the manipulations of the model (e.g., the deletion of FHA/ACT) result in early clearance of B. pertussis in the simulation. Our study of minimal components necessary for early clearance of a secondary infection suggests that Th1RCs along with antibodies can be used as a prophylactic measure. We believe that these and other such observations will be useful for the control of B. pertussis and could be applied to other diseases.

This study addresses the goals of systems biology by effectively encapsulating prior knowledge to generate a mechanistic and predictive understanding at the systems level. Understanding at the network level is necessary for formulating detailed quantitative models of within-host disease dynamics. Our methodology can be extended to other respiratory pathogens, and ultimately to pathogens in general. The outputs of within-host models serve as inputs to broader-scale epidemiological models; for example, the effect of host immune components (intrinsic factors) was shown to be important in cholera epidemics [54,55]. Thus, our study has implications in epidemiological models as well.

## Materials and Methods

### Network assembly and dynamic simulation.

The networks in Figures 1, 3, 4, and 5 were drawn with the SmartDraw software (http://www.smartdraw.com). The dynamic model was implemented by custom Python code.

### Boolean transfer functions.

Here, we explain a few representative logical transfer functions used in our model; a detailed justification of all transfer functions is given in Text S1.

The Boolean rule for activated phagocytic cells is:

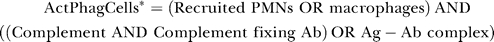
Here, the “AND” operator between phagocytes and antibody-mediated responses describes the fact that phagocytosis requires antigen-specific activation of phagocytes. The “AND” relationship between complement and complement-fixing antibodies denotes that only the classical complement pathway activated through antibodies could activate phagocytes above threshold levels. In case of other infections, the alternative complement pathway also activates phagocytes, but in *Bordetella* infections in B cell–deficient mice, the alternative complement pathway is not sufficient for significant decrease in bacterial numbers [39], supporting the above description. We used an OR relationship between the activation of phagocytes by complement + complement-fixing antibodies and antigen–antibody complexes because PMNs and macrophages are recruited and activated in C3-deficient mice [15,39]. The “OR” condition between PMNs and macrophages is justified by the fact that both of these major cell types contribute directly to the bacterial phagocytosis [56]. These contributions differ in their timing and extent because different activation signals exist for these cell types [57]. Hence, for a simplification in the model, we included separate nodes for PMNs and macrophages and not for each cell type.

The Boolean rules for T helper cells are:

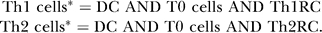
Th cells are activated through the interaction of T0 cells with DCs in the model. Depending on the activation of either Th1RCs or Th2RCs, T0 cells differentiate into Th1 cells or Th2 cells, respectively. The presence of all three components is essential for the activation of a particular T helper cell type; hence, we used an “AND” relationship.

### Parameters.

Our model has two types of parameters. First, the threshold parameters *dc*_*max*_ and *p*_*max*_ signify a condition for the continuous expression of the regulator nodes DCs and Phagocytosis to induce a state change in their respective targets, T0 cells and Bacteria. Second, the decay constants defined for certain antigens (τ*_FHA/ACT_*/τ*_TTSS_*) and cytokines (τ*_Th1RC_*/τ*_Th2RC_*) express the condition that these antigens and cytokines are degraded after being active for more than a given number of time steps, even if the conditions for their activation are still satisfied. Here, we give a biological justification of these parameters.

### Bacterial clearance.

To incorporate the fact that bacteria are not cleared by innate or early adaptive responses, we assume that only a sustained process of phagocytosis is able to clear the infection (i.e., that phagocytosis needs to be continuously on for ***p***_***max***_ time steps).

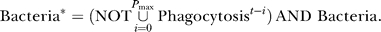
The notation 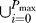 was introduced to indicate that the present (*i* = 0) as well as past (*i* = 1 ⋯ *p*_*max*_) states of the node Phagocytosis compound synergistically to determine the state of the node Bacteria. This condition signifies that only sustained above-threshold levels of phagocytosis lead to bacterial clearance.

### Antigen regulation.

Secreted bacterial toxins and bacterial adhesins such as LPS follow different time courses during pathogenesis. Toxin levels can be reduced by interactions with host immune components (e.g., antigen-specific antibodies) or free decay. Adhesin levels decrease due to a reduction in bacterial numbers or a regulation of the genes encoding the antigens. Though both secreted factors and adhesins can activate antibodies, the removal (neutralization) of the former is not associated with bacterial clearance as they are secreted and released in the host, unlike the latter. We assumed that the levels of PTX are determined by the presence of bacteria and of pertussis-specific antibodies, while O-antigen is expressed constitutively in *B. bronchiseptica.*

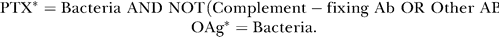



The effect of other virulence factors such as TTSS and FHA/ACT is more complex, possibly due to the cooperation between different virulence factors. The node TTSS expresses the cooperation between TTSS and ACT in *B. bronchiseptica,* and the node FHA/ACT expresses the cooperation between the adhesins FHA, LPS, and the secreted factor ACT in *B. pertussis.* The effects of the TTSS and FHA/ACT are shown to be removed or decreased below threshold levels during late pathogenesis [21,23,27,58]. This reduction could not be reproduced simply by the neutralization of TTSS-secreted factors or of ACT by antibodies. In the absence of detailed information, we assumed that the activity or effect of these nodes decays after a continuous presence for a prespecified number of time steps τ*_TTSS_* and τ*_FHA/ACT_*, even if the conditions that activated them persist. Thus, the transfer function for TTSS is TTSS^*^ = Bacteria AND NOT 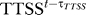 ; similar transfer function applies to FHA/ACT. Thus, the condition for an above-threshold concentration of TTSS or FHA/ACT nodes (state 1) is the presence of their activators at the previous time point, combined with the absence (subthreshold concentration) of TTSS or FHA/ACT τ*_TTSS_* or τ*_FHA/ACT_* time points ago, respectively. Note that it is possible that other virulence factors such as PTX also exhibit uncatalyzed decay/inactivation, but this decay rate is probably smaller than their removal rate by active immune mechanisms. The crucial difference between the effect of secreted factors PTX, on one hand, and ACT and TTSS, on the other hand, is that PTX does not modulate chemokine and cytokine production [23]. Hence, PTX is less likely to have long-lasting effects after its neutralization, unlike ACT and TTSS. Our description of the latter nodes allows us to capture such longer-lasting effects of the nodes by using a decay time longer than one time step.

### Cytokine regulation.

The decision between generating Th1 cells or Th2 cells is determined by a group of cytokines produced during the interaction of antigen-presenting cells and T cells. How the balance between Th1 cells and Th2 cells maintained in the presence of double-negative feedback between Th1-related cytokines and Th2-related cytokines is still unknown. Our modeling of the double-negative feedback between Th1RCs and Th2RCs led to different dynamic outcomes in asynchronous and synchronous algorithms. Asynchronous algorithms gave advantage to the process that is activated earlier (an example of bistability), whereas in the synchronous algorithm synchronous oscillations of Th1RCs and Th2RCs were observed (refer to Table 2 and Text S1 for the Boolean transfer functions of these nodes and to Dynamic simulation for the algorithm). To remove the dependence on the order of update of the asynchronous method in the consensus *Bordetellae* model (without specific virulence factors), we assumed that Th1RCs/Th2RCs decay after a period τ*_Th1RC_*/τ*_Th2RC_*. We found that the value of these decay times is dependent on bacterial virulence factors (refer to Parameter analysis and Text S2 for details). We chose the decay times such that (1) in the species-specific models incorporating TTSS/FHA (in B. bronchiseptica) and FHA/ACT activity (in B. pertussis), a reproducible switch (i.e., activation of Th2RCs followed by Th1RC activation) is observed; and (2) in the consensus model (absent specific virulence factors), there is more than one such switching. Alternative activation of Th1RCs and Th2RCs is found in many disregulations, for example in allergies and allografts [59,60], when the balance between Th1RCs and Th2RCs is not directly modulated, and is also predicted by other models [61].

### Parameter analysis.

In this section we analyze the realistic range of the threshold parameter *p*_*max*_ and the decay constants τ*_TTSS_*/τ*_FHA/ACT_*. All parameters and their analysis are given in Text S2. We performed a systematic search in parameter space to determine the parameter regions that satisfy the following two criteria: (1) reaching bacterial clearance and (2) association of bacterial clearance with activation of phase III. We performed 1,000 simulations for each parameter value in a biologically realistic region. The disease was allowed to evolve for 70 time steps in each simulation. To test the first condition, we determined the distribution of the time steps at which bacteria were cleared; for the second criterion, we monitored the frequency of activation of the node Th1RC around the time step at which bacteria were cleared. Figure S4 shows the evaluation of condition 2 in the case of τ*_TTS_S*, as an example.

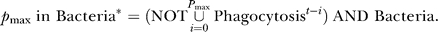
We sampled the threshold for clearance-inducing phagocytosis, *p*_max_, in the interval 0 ≤ *p*_*max*_ ≤ 10. For *p*_*max*_ < 2, B. bronchiseptica were cleared by innate immune responses, which is inconsistent with the literature [2,14,24,39]. For *p*_*max*_ > 1, the bacterial clearance was delayed by one time step for each unit increase in the *p*_max_ value in both species. Increasing *p*_*max*_ above 2 increased the length of phase III, which was shorter in the simulation than in experimental studies. Our perturbation analysis indicated that for *p*_*max*_ > 4, the perturbations in which early clearance was observed (TTSS deletion, antibody-mediated clearance in *B. bronchiseptica;* see Table S3) could not be reproduced, implying that the condition is too stringent. We chose *p*_*max*_ = 4; the success of the results obtained with this value suggest that in later phase III, bacterial concentration might decrease so much that phagocytosis is active at low (close to threshold or even subthreshold) levels, requiring a longer time for complete clearance. Experiments performed at shorter time intervals around the expected clearance time than the customary sampling at days 28, 50, and 70 will be necessary to better elucidate this parameter.

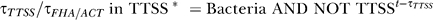
and

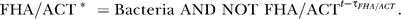
τ_FHA*/ACT*_ and τ*_TTSS_* were varied in the intervals 1 ≤ τ_FHA*/ACT*_ ≤ 22, 1 ≤ τ*_TTSS_* ≤ 22, and the distribution of the time steps of bacterial clearance was plotted (see Figure S4). Clearance was delayed by one time step with each unit increment. When τ*_TTSS_* > 15 or τ*_FHA/ACT_* > 18, bacteria were not cleared. For 3 ≤ τ*_FHA/ACT_* ≤ 8 or 3 ≤ τ*_TTSS_* ≤ 8, the bacterial clearance was not associated with Th1-related responses; for 0 ≤ τ*_FHA/ACT_* or 0 ≤ τ*_TTSS_* ≤ 3, there were spurious oscillations between Th2RCs and Th1RCs. As natural killer cell activation in B. pertussis infection activates IFN-γ (a Th1RC) production [38,62] earlier than in B. bronchiseptica infections, thus τ*_FHA/ACT_* < τ*_TTSS_*. Hence, we used τ*_FHA/ACT_* = 12 and τ*_TTSS_* = 15*.* The result that the composite action of the nodes FHA/ACT and TTSS decreases below threshold levels after 12 and 15 time steps, or after nine to 12 time steps of active antibody response, suggests that these nodes have a longer effect than other independently acting virulence factors such as PTX and O-antigen. A possible mechanism in support of this suggestion is damage to cellular systems; for example, TTSS permeabilizes cell membranes, which require a longer time to heal even after the toxin can be neutralized by antibodies.

### Secondary infections.

Secondary infections were modeled by using initial conditions that represent the state of the host when a secondary bacterial invasion takes place. The secondary initial condition is defined by the active components of the immune system and bacteria. Three scenarios were simulated. First, an infection of a diseased host was represented using an initial condition where the nodes “Epithelial cells,” “Ag–Ab complex,” “Complement-fixing Ab,” “Other Ab,” “Th2RCs,” “T0 cells,” “Th2 cells,” and “DCs” were active. The node “Complement” was also active in the case of B. pertussis infection. Second, the infection of a convalescent host was represented by an initial condition in which the following nodes were on: “Epithelial cells,” “Complement,” “Ag–Ab complex,” “Complement-fixing Ab,” “Other Ab,” “B cells,” “Th1RCs,” “PICs,” “T0 cells,” “Th1 cells,” “Recruited PMNs,” “Macrophages,” and “DCs.” Last, the simulation of infection of immune hosts was performed by using the memory steady state of primary infections as initial condition (i.e., setting the two antibody nodes “Complement-fixing Ab” and “Other Ab” on, along with the node “Bacteria”).

### Bacteria.

We used the sequenced WT strains of B. bronchiseptica (RB50) and B. pertussis (BP536) as described previously [63,64]. Bacteria were maintained on Bordet-Gengou agar (Difco, http://www.bd.com), inoculated into Stainer-Scholte broth at optical densities of 0.1 or lower, and grown to mid-log phase at 37 °C on a roller drum.

### Animal experiments.

C57BL/6 mice were obtained from The Jackson Laboratory (http://www.jax.org). Mice were maintained and treated at the Pennsylvania State University in accordance with approved institutional guidelines. Prior to inoculation, mice were lightly sedated with isoflurane (Abbott Laboratories, http://www.abbott.com) and were inoculated by pipetting 50 μl of phosphate-buffered saline containing the 5 × 10^5^ CFU of B. bronchiseptica or B. pertussis onto the tip of the external nares. For bacterial enumeration, groups of four animals were killed at the indicated time point after inoculation. Colonization of respiratory organs was quantified by homogenization of each tissue in phosphate-buffered saline, plating onto Bordet-Gengou blood agar containing 20 μg/ml streptomycin, and colony counting. For reinfection experiments, mice were intranasally inoculated with 5 × 10^5^ CFU of bacteria in 50 μL and reinfected with the same dose 28 d after the initial inoculation. Mice were dissected 3 d after the reinfection, and bacterial numbers were enumerated as described above.

## Supporting Information

Figure S1 Experimental Time Courses of IFN-γ and PMNs (A) For the IFN-γ time course, groups of three to four C57BL/6 mice were intranasally inoculated with 5 × 10^5^ CFU of the WT (filled diamonds) or ΔbscN (open squares) strain of *B. bronchiseptica.* The ΔbscN strain lacks the ATPase required for the secretion of TTSS. The splenocytes were collected at the indicated time points after inoculation and restimulated for 3 d with heat-killed *B. bronchiseptica,* and the supernatant was analyzed for IFN-γ by ELISA.
(B) For the PMN time course, C57BL/6 mice were inoculated with 5 × 10^5^ CFU of B. pertussis (circles) or B. pertussis ΔPTX (squares). Mice were killed on the indicated days, their lungs were perfused with 5 ml cold phosphate-buffered saline, and total leukocytes per whole lung were enumerated. Individual cell types were determined by modified Giemsa staining of cells. *n* = 4 per group. *p < 0.001; **p < 0.05.
(17 KB PDF)

Figure S2 Distributions of B. bronchiseptica and B. pertussis Clearance upon Implementation of Two Constraints on the Order of Updates (A) Epithelial cells updated before dendritic cells.
(B) Th1RCs are updated before Th2RCs, and Th2RCs are updated before PICs.
(408 KB AI).

Figure S3 B. bronchiseptica (Bb) and B. pertussis (Bp) Clearance Time Distributions in the Completely Asynchronous Model for the Following Perturbations (A) TTSS deletion or FHA/ACT deletion.
(B) Th1 cell deletion.
(C) Macrophage deletion.
(D) Antibody treatment.
(661 KB TIF)

Figure S4 Parameter Analysis (A) The frequency of an association between bacterial clearance and the activity of Th1RCs as a function of the parameter τ*_TTSS_*. Similar frequency plots were made for all other parameters in the simulation.
(B) The dependence of the timing of bacterial clearance on the parameters τ*_TTSS_* in *B*. *bronchiseptica* (gray) and τ*_FHA/ACT_* in B. pertussis (green). All symbols represent 100% of the clearance times when the simulation was run for 1,000 times for each τ*_TTSS_* and τ*_FHA/ACT_*.
(C) Oscillations of Th1RCs (blue) and Th2RCs (purple) in B. pertussis for τ*_Th1RC_* = τ*_Th2RC_* = 1. The corresponding time course in B. bronchiseptica is identical with the exception that Th1RCs are activated on the fifth time step.
(637 KB TIF)

Table S1 Synthesis of Experimental Information about Regulatory Interactions between Host Immune Components and Bacterial Virulence Factors (97 KB DOC)

Table S2 Temporal Information Known from Experimental Observations in B. bronchiseptica (Bb) and B. pertussis (Bp) (66 KB DOC)

Table S3 Summary of Experimental Data on the Effect of Bacteria/Mouse Knockout Mutations on Bacterial Clearance This information was used to test the model's outcomes in the corresponding simulated knockouts. For comparison, the timing of the WT infections is also given.
(40 KB DOC)

Table S4 Nodes Active in (a Portion of) Each Phase of the Infection with B. bronchiseptica (Bb) and B. pertussis (Bp) (55 KB DOC)

Table S5 B. bronchiseptica and B. pertussis Clearance Time Steps during Simulated Cross-Infections (29 KB DOC)

Text S1 Detailed Explanation of Boolean Transfer Functions (85 KB DOC)

Text S2 Parameter Analysis (90 KB DOC)

Text S3 Cross-Infections (27 KB DOC)

### Accession Numbers

The GeneDB (http://www.genedb.org) accession numbers for the bacterial genes discussed in this paper are: adenylate cyclase toxin (BB0324 in B. bronchiseptica, BP0760 in B. pertussis), filamentous hemagglutinin (BB2993 in B. bronchiseptica, BP1879 in B. pertussis), pertussis toxin (five subunits: BP3786, BP3785, BP3787, BP3783, BP3784), and type III secretion system (BB1628). The ExPASy database (http://ca.expasy.org) accession number for Toll-like receptor 4 is TLR4_HUMAN (O00206) and TLR4_MOUSE (Q9QUK6).

## Abbreviations

ACT: adenylate cyclase toxin
DC: dendritic cell
FHA: filamentous hemagglutinin
LPS: lipopolysaccharide
PIC: proinflammatory cytokine
PMN: polymorphonuclear leukocyte
PTX: pertussis toxin
Th: T helper type
Th1RC: Th1-related cytokine
TLR4: Toll-like receptor 4
TTSS: type III secretion system
