# Supplementary material for: Modeling Systems-Level Regulation of Host Immune Responses
Source: PLoS Comput Biol. 2007 Jun 8;3(6):e109. doi: 10.1371/journal.pcbi.0030109 (PMC1892604; doi:10.1371/journal.pcbi.0030109)
Supplement: Supplementary file 1 [file pcbi.0030109.sg001.pdf]

(A)

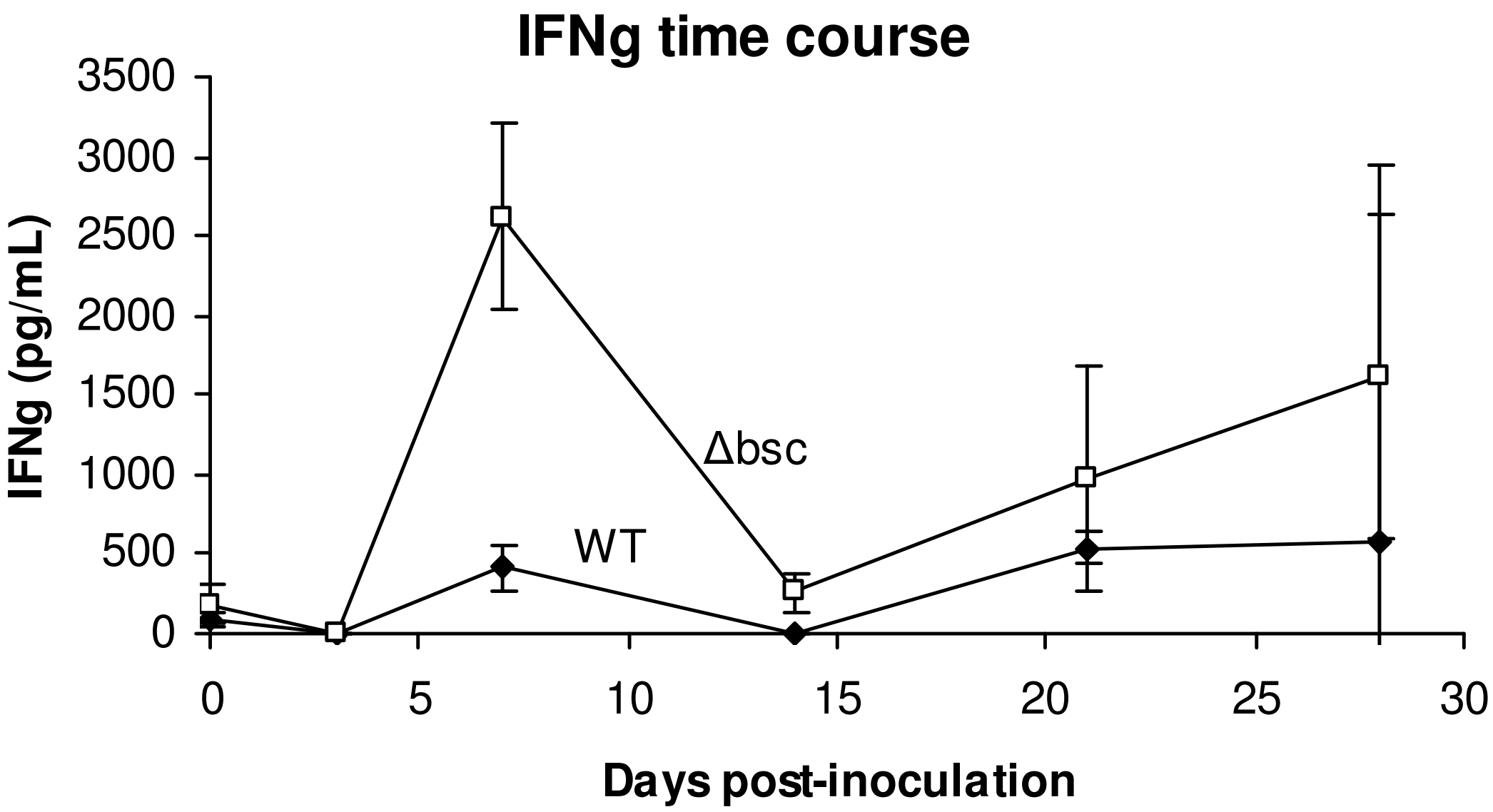
IFNg time course
IFNg (pg/mL)
3500
3000
2500
2000
1500
1000
500
0
0
5
10
15
20
25
30
Days post-inoculation
Δbsc
WT

(B)

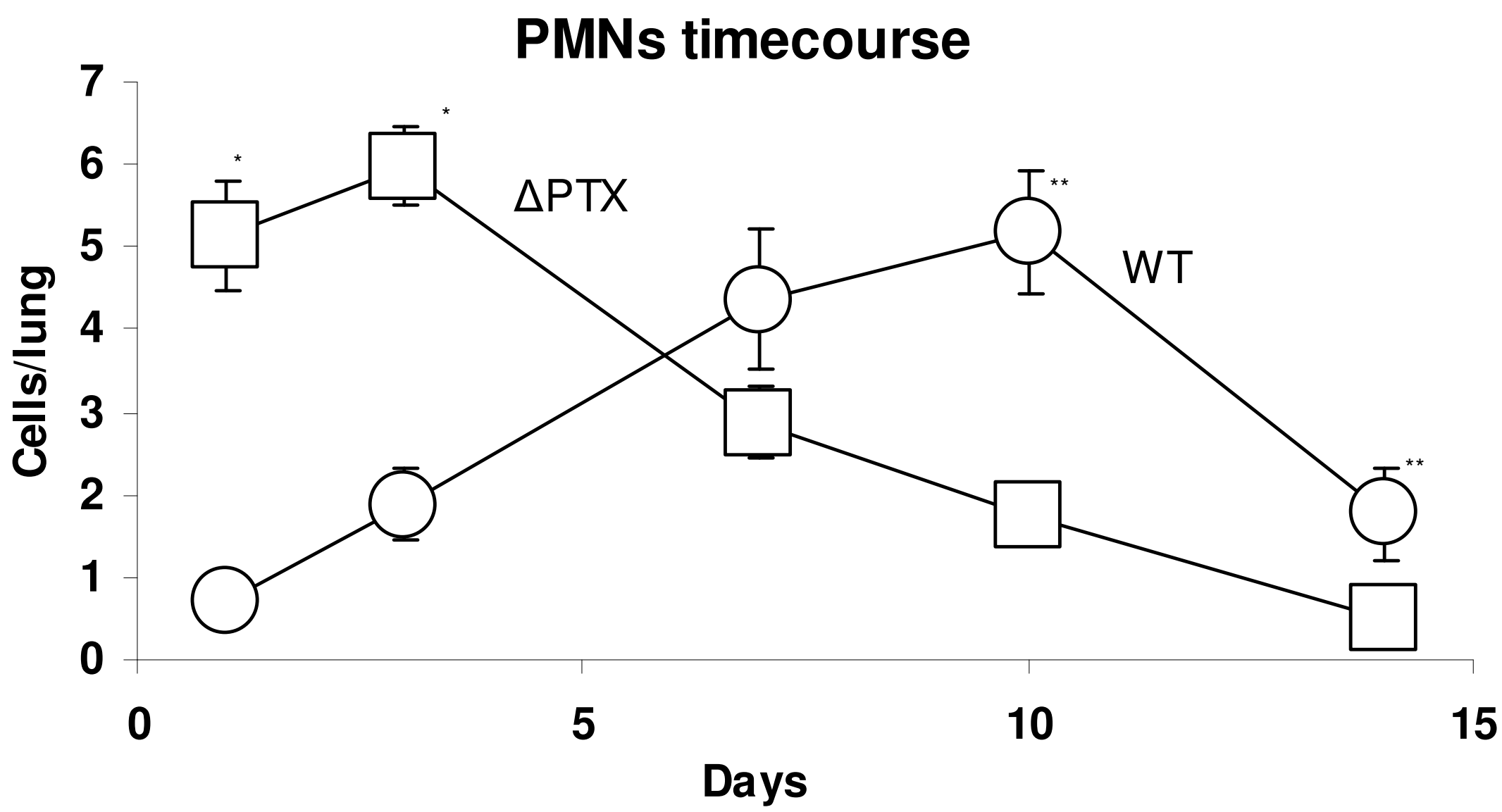
PMNs timecourse
Cells/lung
7
6
5
4
3
2
1
0
0
5
10
15
Days
ΔPTX
WT
*
*
**
**
