# Supplementary material for: Modeling Systems-Level Regulation of Host Immune Responses
Source: PLoS Comput Biol. 2007 Jun 8;3(6):e109. doi: 10.1371/journal.pcbi.0030109 (PMC1892604; doi:10.1371/journal.pcbi.0030109)
Supplement: Supplementary file 2 [file pcbi.0030109.sg002.pdf]

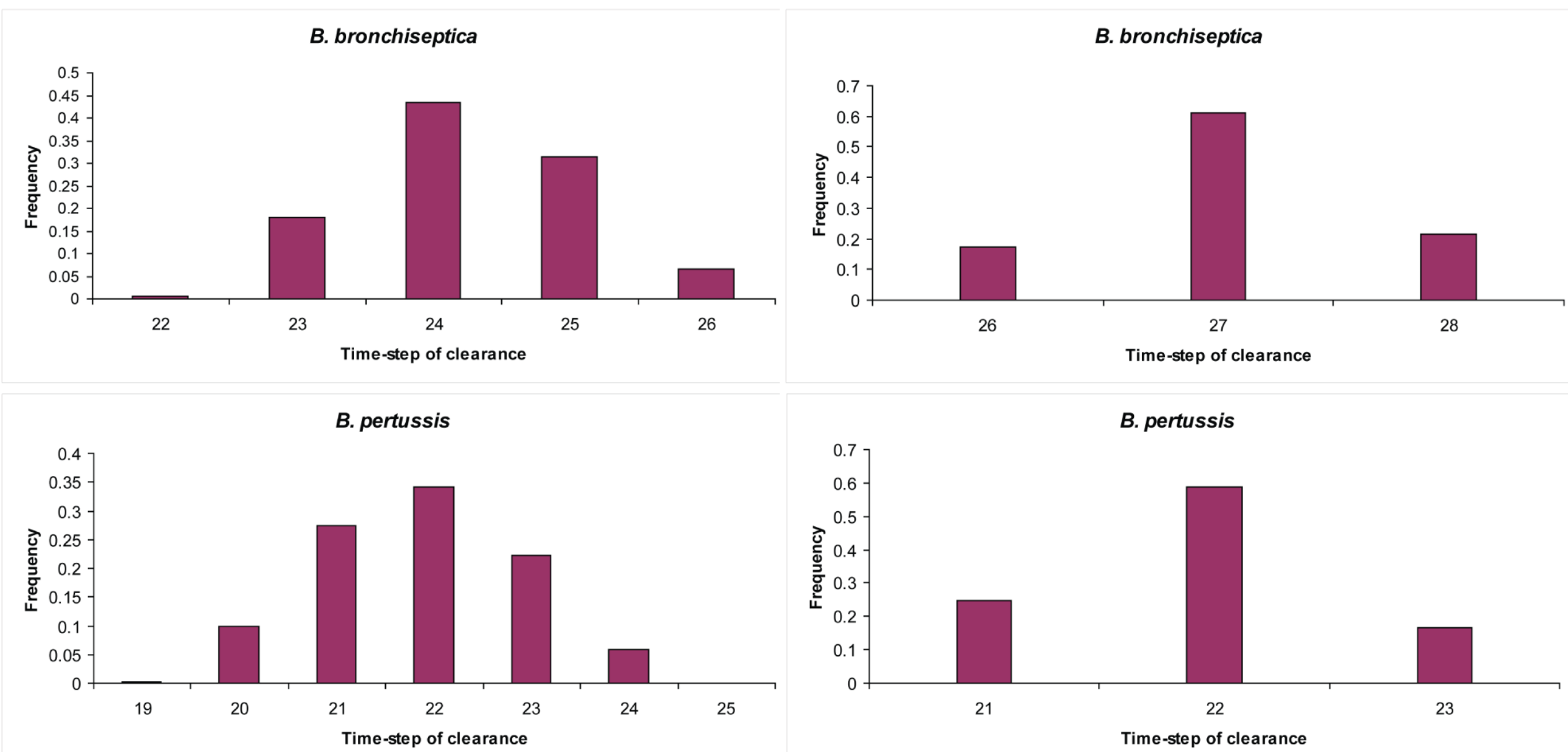
B. bronchiseptica
Frequency
0.5
0.45
0.4
0.35
0.3
0.25
0.2
0.15
0.1
0.05
0
22
23
24
25
26
Time-step of clearance
B. bronchiseptica
Frequency
0.7
0.6
0.5
0.4
0.3
0.2
0.1
0
26
27
28
Time-step of clearance
B. pertussis
Frequency
0.4
0.35
0.3
0.25
0.2
0.15
0.1
0.05
0
19
20
21
22
23
24
25
Time-step of clearance
B. pertussis
Frequency
0.7
0.6
0.5
0.4
0.3
0.2
0.1
0
21
22
23
Time-step of clearance
