# Supplementary material for: Modeling Systems-Level Regulation of Host Immune Responses
Source: PLoS Comput Biol. 2007 Jun 8;3(6):e109. doi: 10.1371/journal.pcbi.0030109 (PMC1892604; doi:10.1371/journal.pcbi.0030109)
Supplement: Supplementary file 3 [file pcbi.0030109.sd001.doc]

**Supplementary text 1: Detailed explanation of Boolean transfer functions**

**Shorthand notations used in the Boolean rules:**

PIC = Pro-inflammatory cytokines

PMNs = polymorphonuclear leukocytes

Th1RC = Th1 related cytokines

Th2RC = Th2 related cytokines

Ab = Antibodies

Ag = Antigen

ActPhagCells = Activated phagocytic cells

DC = Dendritic cells

TTSS = Type three secretion system

PTX = Pertussis toxin

FHA/ACT = Composite action of Filamentous Hemagluttinin and Adenylate Cyclase Toxin

The * subscript indicates the future state of the respective node.

**Epithelial cells^*^ = Bacteria**

**DC* = Bacteria AND (TH1RC OR Th2RC OR PIC)**

Epithelial cells and dendritic cells are the two cell types in the present model which can directly respond to bacteria. Epithelial cells lining the inside of the lungs are the first ones to come across the pathogens. Hence their activation state directly depends on the presence or absence of the bacteria. Toll-like receptors (TLR4) on epithelial cells recognize antigens and induce pro-inflammatory cytokines.

Dendritic cells are present in small quantities in tissues in the inner lining of the lungs in immature state. This state is characterized by high endocytic activity and low T cell activation potential. The rule indicates that dendritic cells are activated by above-threshold levels of bacteria together with any of the three types of cytokines (denoted by the OR operator between the three). The AND condition between bacteria and cytokines makes sure that dendritic cells and in turn the adaptive immunity is not activated in the absence of bacteria.

**PIC* = Epithelial cells OR ActPhagCells AND NOT Th2RC**

Epithelial cells are the first ones to produce pro-inflammatory cytokines [1]. But when phagocytes (denoted ActPhagCells) are activated, they also produce pro-inflammatory cytokines. We assume that either cell type produces an above threshold level of pro-inflammatory cytokines, and combine the two processes by an OR relationship. Many Th2 related cytokines are anti-inflammatory; we assume that they effectively block the production of pro-inflammatory cytokines, expressed by the AND NOT operator.

**Recruited PMNs* = PIC**

**Macrophages* = PIC OR Th1RC**

Macrophages and recruited polymorphonuclear leukocytes (PMNs) are the major phagocytes activated during the invasion by pathogen. Their contributions differ in their timing and extent as the activation signals for these cell types can be different [2]. Hence for simplicity in the model we included separate nodes for PMNs and macrophages and not for each cell type. Pro-inflammatory cytokines recruit PMNs and macrophages. In addition to pro-inflammatory cytokines, Th1 related cytokines can also activate macrophages, and we assume that there is no conditional dependence between the two classes of signals.

**Complement* =Bacteria OR (Ag-Ab complex AND Complement fixing Ab)**

The complement system can be activated by either the alternative or the classical complement pathway. The alternative complement pathway is activated directly via recognition of bacterial virulence factors. The activation of classical complement pathway require the antigens bound with complement fixing antibodies (e.g. IgG, IgM), accounted for by the AND operator between the Ag-Ab complex and complement fixing Ab.

**Complement fixing Ab* = B cells OR Complement fixing Ab**

**Other Ab* =B cells OR Other Ab**

**Ag-Ab complex* =Bacteria AND (Other Ab OR Complement fixing Ab)**

Complement fixing antibodies (e.g. IgG, IgM) and other types of antibodies (e.g. IgE) take part in different processes, hence we represented them with two separate nodes based on basic immunological understanding. Antibodies (complement fixing or otherwise) are secreted from B cells. As we know that antibodies once activated are sustained for longer time, ultimately contributing to immunological memory, we assume that the future state of antibodies depends positively on their present state. The OR relationship between B cells and antibodies expresses the assumption that once produced, antibodies do not decay completely and is equivalent with a positive feedback loop, or memory, on the antibody nodes. Antigen-antibody complex formation is indicated as an AND relationship between antigens and antibodies. The OR operator between different types of antibodies represents that both types of antibodies can form the complex.

**ActPhagCells* = (Recruited PMNs OR macrophages) AND ((Complement AND Complement fixing Ab) OR Ag-Ab complex))**

The ‘AND’ operator between phagocytes (PMNs and macrophages) and antibody mediated responses describes that phagocytosis requires both the presence, and antigen-specific activation, of phagocytes. The ‘AND’ relationship between complement and complement fixing antibodies denotes the assumption that among complement system induced mechanisms acting on phagocytes, only the classical complement pathway activated through antibodies can activate phagocytes above threshold levels. In case of other infections the alternative complement pathway also activates phagocytes but in Bordetella infections the alternative complement pathway is not sufficient for significant decrease in bacterial numbers in B cell deficient mice [3], supporting the above description. We used an OR relationship between the activation of phagocytes by complement + complement fixing antibodies, and antigen-antibody complexes, because PMNs and macrophages are recruited and activated in complement (C3) deficient mice [3,4]. The ‘OR’ condition between PMNs and macrophages is justified by the fact that both of these major cell types contribute directly to bacterial phagocytosis [5].

$$\text{ T0 cells}=\underset{i=1}{\overset{\text{dc}_{\text{max}}}{}}\text{ DC}^{\text{t-i}}$$

**Th1 cells^*^ = DC AND T0 cells AND Th1RC**

**Th2 cells^*^ = DC AND T0 cells AND Th2RC**

T0 cells are activated through their interaction with dendritic cells (and antigen presenting cells in general). Activated dendritic cells migrate to the lymphoid tissues where they interact with T0 cells. To include the delay due to dendritic cell migration and to incorporate the assumption that a threshold number of dendritic cells will be necessary to induce the activation of adaptive immune responses, we included a threshold parameter *dc_max_*. The notation$\underset{i=1}{\overset{\text{dc}_{\text{max}}}{}}\text{DC}^{\text{t-i}}$signifies that T0 cells are activated only if the node dendritic cells is active for *dc_max_* timesteps (see Supplementary text 2 for parameter analysis of *dc_max_*). T0 cells differentiate into T helper cells through the interaction of T0 cells with DCs and with cytokines in the model. Depending on the presence of either Th1 related cytokines (Th1RC) or Th2 related cytokines (Th2RC), T0 cells differentiate into Th1 cells or Th2 cells respectively. The presence of all three components is essential for the proliferation of a particular T helper cell type, hence we used an ‘AND’ relationship.

**B cells* =Th2 Cells**

B cells’ clonal expansion is stimulated through their interaction with Th2 cells. Hence the state of the node B cells follows the state of Th2 cells with a delay.

**Th1RC* = (Th1 cells OR (DC AND T0 cells)) AND NOT Th2RC AND NOT Th1RC**${}^{t-\tau_{\text{Th}1\text{RC}}}$

**Th2RC* = (Th2 cells OR (DC AND T0 cells)) AND NOT Th1RC AND NOT Th2RC**${}^{t-\tau_{\text{Th}2\text{RC}}}$

The equation represents that Th1/Th2 related cytokines can be produced by Th1/Th2 cells or by T0 cells (naïve T cells) during their interaction with dendritic cells (antigen presenting cells). Th1/Th2 related cytokines inhibit Th2/Th1 related cytokines, shown by the first AND NOT operator. How the balance between Th1 cells and Th2 cells is maintained in the presence of cross-inhibition between Th1 related cytokines (Th1RC) and Th2 related cytokines (Th2RC) is still unknown. Our modeling of this cross-inhibition between Th1RC and Th2RC, and the positive feedback loop caused by it, led to different dynamic outcomes in asynchronous and synchronous algorithms. Asynchronous algorithms gave advantage to the process that is activated earlier (an example of bistability), whereas in the synchronous algorithm synchronous oscillations of Th1RC and Th2RC were observed (see the section Dynamic simulation for a description of the synchronous algorithm). To remove the dependence on the order of update of the asynchronous method in the consensus bordetellae model (the model without specific virulence factors), we assumed that Th1 / Th2 related cytokines decay after a period *τ_Th1RC_* / *τ_Th2RC_* (refer to Supplementary text 2 for parameter analysis for *τ_Th1RC_* / *τ_Th2RC_* ). This is represented by the second AND NOT operator, indicating that Th1RC/Th2RC are degraded after continuous presence for *τ_Th1RC_* / *τ_Th2RC_* timesteps.

**Phagocytosis* = ActPhagCells AND Bacteria**

Phagocytosis is the output node in our network and is active if both activated phagocytic cells (ActPhagCells) and Bacteria are on (i.e. bacteria are phagocytosed by activated phagocytic cells when they are present).

$$\text{Bacteria}\text{ }=(\text{NOT }\underset{i=0}{\overset{p_{\text{max}}}{\cup}}\text{Phagocytosis}^{\text{t-i}})\text{ AND Bacteria}$$

Bacterial levels depend on bacterial division and the intensity of phagocytosis. Thus the node bacteria is active if there is no phagocytosis and if there are bacteria at time t-t_b_ , where t_b_ is the time necessary to increase the bacterial concentration to the threshold (on) level. The condition ‘AND Bacteria’ makes sure that if there is no bacterial invasion the node is off even when there is no phagocytosis. We assume that only sustained above-threshold levels of phagocytosis, i.e. the ON state of the node phagocytosis for *p_max_* timesteps, are sufficient for bacterial clearance. The notation $\underset{i=0}{\overset{p_{\text{max}}}{}}\text{Phagocytosis}^{\text{t-i}}$ was introduced to indicate that the present (i=0) as well as past (i=1.. *p_max_* ) states of the node phagocytosis compound synergistically to determine the state of the node bacteria.

Species specific nodes are described for *B. bronchiseptica* and *B. pertussis* below.

***B. bronchiseptica***

$$\text{TTSS}=\text{Bacteria AND NOT TTSS}^{t-\tau_{\text{TTSS}}}$$

**Dead PMNs* = Recruited PMNs AND TTSS**

The bacterial type three secretion system (TTSS) is activated at or closely following inoculation. The node TTSS expresses the co-operation between TTSS and ACT. The effect of TTSS is shown to be removed or decreased below threshold levels during late pathogenesis [6]. This reduction could not be reproduced simply by assuming TTSS’s neutralization by antibodies. In the absence of detailed information, we assumed that the activity or effect of TTSS decays after a continuous presence for a pre-specified number of timesteps *τ_TTSS_* even if the conditions that activated it persist. Thus the node is active only if node Bacteria is on and the node TTSS has not been on for more than *τ_TTSS_* timesteps (see Supplementary text 2 for parameter analysis of *τ_TTSS_*). TTSS induce necrosis in PMNs recruited to the site of infection [7] , which we indicate by defining a node Dead PMNs that is on if both Recruited PMNs and TTSS nodes are on.

**Th1 cells* = (DC AND T0 cells AND Th1RC) AND NOT TTSS**

**Th1RC* = (Th1 cells OR (DC AND T0 cells)) AND NOT Th2RC AND NOT Th1RC**${}^{t-\tau_{\text{Th}1\text{RC}}}$ **AND NOT TTSS**

The equations differ from the general rules described earlier in incorporating the effect of the bacterial type III secretion system (TTSS) on the differentiation of T0 cells and the production of Th1 related cytokines. We assume that TTSS blocks the differentiation into Th1 cells, shown by the AND NOT operator in the Boolean rule for Th1 cells. TTSS also affects the differentiation into Th1 cells by inhibiting the production of Th1RC *B. bronchiseptica* [8], shown by the last AND NOT operator.

**Complement* = (Bacteria AND NOT OAg) OR (Ag-Ab complex AND Complement fixing Ab)**

**OAg* = Bacteria**

The O antigen is an adhesion factor expressed on the surface of bacteria. As it is not secreted like toxins, opsonization of bacteria and deactivation of OAg was assumed to be equivalent processes. The alternative complement pathway is activated directly via recognition of bacterial virulence factors and this pathway is inhibited by *B. bronchiseptica* O-antigen [9]. The OR relationship in the Boolean rule for complement is used to show the activation of complement by the alternative or classical complement pathways. The AND operator between Ag-Ab complex and Complement fixing Ab represent that the activation of classical complement pathway require the antigens bound with complement fixing antibodies e.g. IgG, IgM.

***B. pertussis***

**Recruited PMNs* = PIC AND NOT PTX**

**PTX* = Bacteria AND NOT (Complement fixing Ab OR Other Ab)**

Secreted bacterial toxin levels can be reduced by interactions with host immune components (e.g. antigen-specific antibodies) or free decay. The removal (neutralization) of the secreted PTX toxin is not associated with and is thought to precede bacterial clearance [10]. Hence we assumed that the levels of pertussis toxin are determined by the presence of bacteria and of pertussis-specific antibodies, which could be either complement-fixing or not. The pertussis toxin is known to inhibit recruitment of PMNs [10], hence in addition to the general conditions for recruitment of PMNs, an AND NOT operator is included to describe the inhibition by PTX.

**Th1RC* = (Th1 cells OR (DC AND T0 cells)) AND NOT Th2RC AND NOT Th1RC AND NOT FHA/ACT**

**FHA/ACT* = Bacteria AND NOT FHA/ACT**${}^{t-\tau_{\text{FHA}/\text{ACT}}}$

The node FHA/ACT expresses the co-operation between the adhesins Filamentous hemaglutinnin (FHA), lipopolysaccharide (LPS) and the secreted factor Adenylate cyclase toxin (ACT). The equation represents that FHA/ACT is activated by the node Bacteria. The effects of FHA/ACT are shown to be removed or decreased below threshold levels during late pathogenesis [11]. This reduction could not be reproduced simply by assuming FHA/ACT’s neutralization by antibodies. In the absence of detailed information, we assumed that the activity or effect of FHA/ACT decays after a continuous presence for a pre-specified number of timesteps *τ_FHA/ACT_* even if the conditions that activated it persist Thus the node is active only if node Bacteria is on and the node FHA/ACT has not been on for more than *τ_FHA/ACT_* timesteps (see Supplementary text 2 for parameter analysis of *τ_FHA/ACT_*). In addition to the general Th1RC rule described earlier, we included the last AND NOT operator to represent the inhibition of Th1 related cytokines by the two virulence factors combined in one node FHA/ACT in *B. pertussis* [11]. It is also known that LPS assist in this inhibition indicating the complexity of the process. The actual role of individual virulence factor is not known in this inhibition.

**References**

1. Mann PB, Elder KD, Kennett MJ, Harvill ET (2004) Toll-like receptor 4-dependent early elicited tumor necrosis factor alpha expression is critical for innate host defense against Bordetella bronchiseptica. Infect Immun 72: 6650-6658.

2. Nomoto K (1998) Role of phagocytosis by PMN and macrophages in bio-defense. Nippon Saikingaku Zaashi 43: 899-910.

3. Kirimanjeswara GS, Mann PB, Harvill ET (2003) Role of antibodies in immunity to Bordetella infections. Infect Immun 71: 1719-1724.

4. Pishko EJ, Kirimanjeswara GS, Pilione MR, Gopinathan L, Kennett MJ, et al. (2004) Antibody-mediated bacterial clearance from the lower respiratory tract of mice requires complement component C3. Eur J Immunol 34: 184-193.

5. Stossel TP (1977) Phagocytosis. Prog Clin Biol Res 13: 87-102.

6. Skinner JA, Pilione MR, Shen H, Harvill ET, Yuk MH (2005) Bordetella type III secretion modulates dendritic cell migration resulting in immunosuppression and bacterial persistence. J Immunol 175: 4647-4652.

7. Yuk MH, Harvill ET, Cotter PA, Miller JF (2000) Modulation of host immune responses, induction of apoptosis and inhibition of NF-kappaB activation by the Bordetella type III secretion system. Mol Microbiol 35: 991-1004.

8. Pilione MR, Harvill ET (2006) The Bordetella bronchiseptica type III secretion system inhibits gamma interferon production that is required for efficient antibody-mediated bacterial clearance. Infect Immun 74: 1043-1049.

9. Burns VC, Pishko EJ, Preston A, Maskell DJ, Harvill ET (2003) Role of Bordetella O antigen in respiratory tract infection. Infect Immun 71: 86-94.

10. Kirimanjeswara GS, Agosto LM, Kennett MJ, Bjornstad ON, Harvill ET (2005) Pertussis toxin inhibits neutrophil recruitment to delay antibody-mediated clearance of Bordetella pertussis. J Clin Invest 115: 3594-3601.

11. Boyd AP, Ross PJ, Conroy H, Mahon N, Lavelle EC, et al. (2005) Bordetella pertussis Adenylate Cyclase Toxin Modulates Innate and Adaptive Immune Responses: Distinct Roles for Acylation and Enzymatic Activity in Immunomodulation and Cell Death. J Immunol 175: 730-738.
