# Supplementary material for: Modeling Systems-Level Regulation of Host Immune Responses
Source: PLoS Comput Biol. 2007 Jun 8;3(6):e109. doi: 10.1371/journal.pcbi.0030109 (PMC1892604; doi:10.1371/journal.pcbi.0030109)
Supplement: Supplementary file 4 [file pcbi.0030109.sd002.doc]

**Supplementary text 2: Parameter analysis**

The model has two types of parameters. First, the threshold parameters *dc_max_* and *p_max_*, denote a condition for the continuous expression of the regulator nodes dendritic cells and phagocytosis to induce a state change in their respective targets, T0 cells and bacteria. Second, decay constants defined for certain antigens and cytokines express the condition that they are degraded after being active for more that a given number of time-steps even if the conditions for their activation are still satisfied. The rationale for the first type of parameters is that the expressions of certain target nodes require higher expression or activity level, or sustained activity, of their source nodes. This phenomenon is often found in the regulation of biological signaling pathways, and can be achieved by convergent regulatory motifs like feed-forward loops and positive feed-back loops [4]. Although our model includes all important components of the immune system, there clearly are additional cytokines, signal transduction pathways and cell types that may be responsible for these regulatory motifs. Our present network and parameter setup makes it possible to identify the sub-networks where a more detailed representation is required. The second type of parameters is again very commonly used in modeling biological signaling. In the present model these parameters indicate that the antigens or cytokines are degraded; further experimental analysis will be necessary to determine whether there is a free decay or inhibition due to immune response. The null value of the threshold parameters is zero (i.e. no requirement for sustained activation) and the null value of the decay constants is infinity (i.e. activity only decays if the activators are not expressed anymore).

We performed a systematic search in parameter space, starting from the null values and only using a non-null value when necessary. The minimal criterion for accepting a parameter value was i) reaching bacterial clearance and ii) association of bacterial clearance with activation of phase III in a simulation of a wild type infection. We performed 1000 simulations for each parameter value in a biologically realistic region. The disease was allowed to evolve for 70 time-steps in each simulation. To test the first condition we determined the distribution of the time-steps at which bacteria were cleared and for the second criterion we monitored the frequency of activation of the node Th1RC around the time-step at which bacteria were cleared. Figure S4 shows the evaluation of the second condition in case of *τ_TTSS_* as an example**.** Parameters satisfying the minimal conditions set by the wild type model were further filtered by analyzing network perturbations.

**Threshold parameters:**

***p_max_***	in	$\text{Bacteria}\text{ }=(\text{NOT }\underset{i=0}{\overset{p_{\text{max}}}{\cup}}\text{Phagocytosis}^{i})\text{ AND Bacteria}$

The requirement for this threshold was one of the first outcomes of the model as it is known from the Bordetella pathogenesis that clearance from the lower respiratory tract is due to an adaptive immune response. Since phagocytic cells and phagocytosis are activated during the innate immune response (phase I, see Table S2), this condition is needed to ensure that innate immunity (phase I) is not sufficient for the clearance of bacteria. We sampled the threshold for clearance-inducing phagocytosis, *p_max_*, in the interval [0, 10]. For *p_max_<2* *B. bronchiseptica* were cleared by innate immune responses in the simulation, which is inconsistent with the literature [5-8]. For *p_max_ > 1*, the bacterial clearance was delayed by one time-step for each unit increase in the *p_max_* value in both species. Increasing *p_max_* above 2 increased the length of phase III, which was shorter in the simulation results as compared the one found in experimental studies. Our perturbation analysis indicated that for *p_max_ > 4* the perturbations in which early clearance was observed (TTSS deletion, antibody mediated clearance in *B. bronchiseptica*, see Table S3) could not be reproduced, implying that the condition is too stringent. We chose *p_max_ = 4*; the success of the results obtained with this value suggest that in late phase III bacterial concentration might decrease so much that phagocytosis is active at low (close to threshold or even sub-threshold) levels, requiring longer time for complete clearance. Experiments performed at shorter time intervals around the expected clearance time than the customary sampling at day 28, 50, 70 will be necessary to better elucidate this parameter.

***dc_max_***	in	$\text{T0 Cells}\text{ }=\underset{i=1}{\overset{\text{dc}_{\text{max}}}{}}\text{DC}^{t_{d}- i}$

Activated dendritic cells migrate to the lymphoid tissues where they interact with naive T cells, leading to their differentiation into Th1 or Th2 cells. To include this delay due to dendritic cell migration and to incorporate the assumption that a threshold number of dendritic cells will be necessary to induce their migration and activation of adaptive immune responses, we included a threshold parameter *dc_max_*. We sampled *dc_max_* in the interval [0, 10]. The bacterial clearance was delayed by two time-steps for each unit increase in *dc_max_* , starting from the 24^th^ time-step for *B. bronchiseptica* and 21^nd^ time-step for *B. pertussis* until *dc_max_* = 4. The early clearance of *B. bronchiseptica* in case of prior antibody treatment was not observed for *dc_max_=0* because in these simulations Th2 related cytokines were activated early (at time-step 4), inhibiting phagocyte recruitment. The necessity of a nonzero *dc_max_* in *B. bronchiseptica* indicates that in the presence of antibodies macrophages and PMNs recruited by pro-inflammatory cytokines can clear *B. bronchiseptica*. Nonzero values of *dc_max_* increased the length of phase I leading to the later activation of T helper cell related responses. A *dc_max_* value greater than 4 led to bacterial persistence, indicating that delayed activation of adaptive immune responses leads to bacterial persistence. We used *dc_max_* = 2, indicating a longer time required for the dendritic cell migration and activation of naïve T cells. In *B. pertussis* ACT is known to activate differentiation of T0 cells into Th2 cells earlier upon interaction with dendritic cells [1,9]. As we did not include particular signal transduction pathways in the model, we introduced this effect by setting *dc_max_* to zero in *B. pertussis* infection. Finally, note that having a larger *dc_max_* value in *B. bronchiseptica* than in *B. pertussis* was not the reason for its later clearance compared to *B. pertussis* as even for *dc_max_=0* *B. bronchiseptica* was cleared later than *B. pertussis* (24^th^ time-step versus the 21^th^ time step).

**Decay constants:**

***τ_TTSS_* / *τ_FHA/ACT_*** in **TTSS* = Bacteria AND NOT TTSS**${}^{t-\tau_{\text{TTSS}}}$ and

**FHA/ACT* = Bacteria AND NOT FHA/ACT**${}^{t-\tau_{\text{FHA}/\text{ACT}}}$

The effects of the TTSS and FHA/ACT are shown to be removed or decreased below threshold levels during late pathogenesis [1,3,10,11]. This reduction could not be reproduced simply by the neutralization of TTSS-secreted factors or of ACT by antibodies. In the absence of detailed information, we assumed that the activity or effect of these nodes decays after a continuous presence for a pre-specified number of time-steps *τ_TTSS_* and *τ_FHA/ACT_* even if the conditions that activated them persist. The transfer functions reflect this assumption by referring to the state *τ* time-steps prior to the current state (at t), and require that the corresponding nodes are not expressed at that prior state because that requirement ensures that the node was not continuously present for *τ* time-steps. An infinite decay time (the null assumption) is equivalent with not having the term AND NOT TTSS${}^{t-\tau_{\text{TTSS}}}$.

*τ_FHA/ACT_* and *τ_TTSS_* were varied in the interval [1, 22] and the distribution of the time-steps of bacterial clearance was plotted (see figure S4A, B). Clearance was delayed by one time-step with each unit increment. When *τ_TTSS_ > 15* or *τ_FHA/ACT_ > 18*, bacteria were not cleared. For τ*_FHA/ACT_ / τ_TTSS_* $\in$ *[3, 8]* the bacterial clearance was not associated with Th1 related responses and for *τ_FHA/ACT_ / τ_TTSS_* $\in$ *[0, 3]* there were spurious oscillations between Th2RC and Th1RC. Natural killer (NK) cell activation in *B. pertussis* infection activates IFN- γ (a Th1 related cytokine) production [12,13] earlier than in *B. bronchiseptica* infections, thus *τ_FHA/ACT_ < τ_TTSS_* . Hence we used *τ_FHA/ACT_* = 12 and *τ_TTSS_* = 15*.* The result that the composite action of the nodes FHA/ACT and TTSS decreases below threshold levels after 12 and 15 time-steps, or after 9 to 12 time-steps of active antibody response, suggests that these nodes have a longer lasting effect than other independently acting virulence factors like PTX and O-antigen. A possible mechanism in support of this suggestion is damage to cellular systems, for example TTSS permeabilizes cell membranes, which require a longer time to heal even after the toxin can be neutralized by antibodies.

We did not use a finite (explicit) decay time for the node PTX. It is possible that the secreted pertussis toxin also exhibits uncatalyzed decay/inactivation but this decay rate is probably smaller than its removal rate by active immune mechanisms. The crucial difference between the effect of the secreted factor PTX, on one hand, and ACT and TTSS on the other hand, is that PTX does not modulate chemokine and cytokine production [11]. Hence PTX is less likely to have long-lasting effects after its neutralization, unlike ACT and TTSS. Our description of the latter nodes allows us to capture such longer lasting effects by using a decay time longer than one time-step.

***τ_Th1RC_* _/_ *τ_Th2RC_*** in

**Th1RC* = (Th1 cells OR (DC AND T0 cells)) AND NOT Th2RC AND NOT Th1RC**${}^{t-\tau_{\text{Th}1\text{RC}}}$ **AND NOT TTSS**

**Th1RC* = (Th1 cells OR (DC AND T0 cells)) AND NOT Th2RC AND NOT Th1RC**${}^{t-\tau_{\text{Th}1\text{RC}}}$ **AND NOT FHA/ACT**

How the balance between Th1 cells and Th2 cells is maintained in the presence of cross-inhibition between Th1 related cytokines (Th1RC) and Th2 related cytokines (Th2RC) is still unknown. Asynchronous algorithms give advantage to the process that is activated earlier, which in our case leads to a persistent activation of Th1RC and inhibition of Th2RC. To reproduce both the cross-inhibition between Th1RC and Th2RC and the known involvement of both in the immune response, we assumed that Th1 / Th2 related cytokines decay after a finite period *τ_Th1RC_*, *τ_Th2RC_*. In the **consensus bordetellae model** (without specific virulence factors), when the *τ_Th1RC_*, *τ_Th2RC_* values were smaller than 2, there were spurious cycles of Th1RC and Th2RC (see figure S4C) and Th1 and Th2 related responses did not activate long enough to clear the bacteria, resulting in bacterial persistence For *τ_Th1RC_* and *τ_Th2RC_* >2 bacterial clearance was delayed by one time-step in *B. bronchiseptica* and 2 time-steps in *B. pertussis* for each unit increment of τ. This differential delay was due to the fact that in *B. bronchiseptica* recruited PMNs and macrophages both contribute to the active phagocytes, whereas in *B. pertussis* PMN recruitment is inhibited in the initial stages. As the early clearance of these defective mutants is well established [2], we chose the value *τ_Th1RC_* = *τ_Th2RC_* = 2. Alternative activation of Th1RC and Th2RC is found in many disregulations, for example in allergies and allografts [14,15] when the balance between Th1RC and Th2RC is not directly modulated, and is also predicted by other models [16].

In the **species specific models** incorporating TTSS/FHA (in *B. bronchiseptica)* and FHA/ACT activity (in *B. pertussis),* we chose the decay times such that a reproducible switch, i.e. activation of Th2RC followed by Th1RC activation, is observed. TTSS and FHA/ACT are known to activate Th2RC and inhibit Th1RC production [1,2]. TTSS and FHA/ACT modulation leads to the activation of Th2RC in phase II and due to the positive (i.e. double negative) feedback loop on Th2RC, it would be sustained indefinitely without an explicit decay time. The decay of Th2RC leads to the sub-threshold levels of Th2RC upon decreased response of TTSS and FHA/ACT, leading to the activation of Th1RC in phase III as is observed experimentally [1,2]. Thus we used *τ_Th2RC_* = *τ_TTSS_* = 15 in *B. bronchiseptica* and *τ_Th2RC_* = *τ_FHA/ACT_* = 12 in *B. pertussis* and *τ_Th1RC_* = duration of phase III = 7 because Th1RC is active during the whole phase III [17]. These parameters are dependent of *τ_TTSS_* /*τ_FHA/ACT_* thus we did not analyze them separately.

In conclusion, all of our parameters choices were informed by experimentally observed dynamics in the model. Through the parameter search and adjustment procedure to incorporate observed effects, the model gives insights into temporal constraints in immune regulation. It is evident from the parameter analysis that the parameter values do not need to be fine-tuned, they only need to stay within certain intervals to attain reproducible clearance. Further parameter refinement by follow-up experiments and modeling will increase our understanding about the regulation of immune responses. A model-based understanding is particularly important in immunological studies as experiments are not generally performed on whole pathogens but a subset of virulence factors. We propose this study as the first step to understanding immunological regulation.

**References:**

1. Boyd AP, Ross PJ, Conroy H, Mahon N, Lavelle EC, et al. (2005) Bordetella pertussis Adenylate Cyclase Toxin Modulates Innate and Adaptive Immune Responses: Distinct Roles for Acylation and Enzymatic Activity in Immunomodulation and Cell Death. J Immunol 175: 730-738.

2. Pilione MR, Harvill ET (2006) The Bordetella bronchiseptica type III secretion system inhibits gamma interferon production that is required for efficient antibody-mediated bacterial clearance. Infect Immun 74: 1043-1049.

3. Skinner JA, Pilione MR, Shen H, Harvill ET, Yuk MH (2005) Bordetella type III secretion modulates dendritic cell migration resulting in immunosuppression and bacterial persistence. J Immunol 175: 4647-4652.

4. Alon U (2006) An Introduction to Systems Biology: Design Principles of Biological Circuits; Biology CHCMC, editor. Rehovot, Israel.

5. Kirimanjeswara GS, Mann PB, Harvill ET (2003) Role of antibodies in immunity to Bordetella infections. Infect Immun 71: 1719-1724.

6. Kirimanjeswara GS, Mann PB, Pilione M, Kennett MJ, Harvill ET (2005) The complex mechanism of antibody-mediated clearance of Bordetella from the lungs requires TLR4. J Immunol 175: 7504-7511.

7. McGuirk P, McCann C, Mills KH (2002) Pathogen-specific T regulatory 1 cells induced in the respiratory tract by a bacterial molecule that stimulates interleukin 10 production by dendritic cells: a novel strategy for evasion of protective T helper type 1 responses by Bordetella pertussis. J Exp Med 195: 221-231.

8. Mills KH (2004) Regulatory T cells: friend or foe in immunity to infection? Nat Rev Immunol 4: 841-855.

9. Ross PJ, Lavelle EC, Mills KH, Boyd AP (2004) Adenylate cyclase toxin from Bordetella pertussis synergizes with lipopolysaccharide to promote innate interleukin-10 production and enhances the induction of Th2 and regulatory T cells. Infect Immun 72: 1568-1579.

10. Carbonetti NH, Artamonova GV, Mays RM, Worthington ZE (2003) Pertussis toxin plays an early role in respiratory tract colonization by Bordetella pertussis. Infect Immun 71: 6358-6366.

11. Kirimanjeswara GS, Agosto LM, Kennett MJ, Bjornstad ON, Harvill ET (2005) Pertussis toxin inhibits neutrophil recruitment to delay antibody-mediated clearance of Bordetella pertussis. J Clin Invest 115: 3594-3601.

12. Byrne P, McGuirk P, Todryk S, Mills KH (2004) Depletion of NK cells results in disseminating lethal infection with Bordetella pertussis associated with a reduction of antigen-specific Th1 and enhancement of Th2, but not Tr1 cells. Eur J Immunol 34: 2579-2588.

13. Della Chiesa M, Sivori S, Castriconi R, Marcenaro E, Moretta A (2005) Pathogen-induced private conversations between natural killer and dendritic cells. Trends Microbiol 13: 128-136.

14. Salem ML (2004) Estrogen, a double-edged sword: modulation of TH1- and TH2-mediated inflammations by differential regulation of TH1/TH2 cytokine production. Curr Drug Targets Inflamm Allergy 3: 97-104.

15. Chan CC, Stark J, George AJ (1999) Analysis of cytokine dynamics in corneal allograft rejection. Proc Biol Sci 266: 2217-2223.

16. Yates A, Bergmann C, Van Hemmen JL, Stark J, Callard R (2000) Cytokine-modulated regulation of helper T cell populations. J Theor Biol 206: 539-560.

17. Mills KH, Barnard A, Watkins J, Redhead K (1993) Cell-mediated immunity to Bordetella pertussis: role of Th1 cells in bacterial clearance in a murine respiratory infection model. Infect Immun 61: 399-410.
