# Supplementary material for: Modeling Systems-Level Regulation of Host Immune Responses
Source: PLoS Comput Biol. 2007 Jun 8;3(6):e109. doi: 10.1371/journal.pcbi.0030109 (PMC1892604; doi:10.1371/journal.pcbi.0030109)
Supplement: Supplementary file 5 [file pcbi.0030109.sd003.doc]

**Supplementary text 3: Cross-infections**

We simulated *B. pertussis* (Bp) after *B. bronchiseptica* (Bb) and *B. bronchiseptica* after *B. pertussis* cross-infections and summarize the results in Table S5. We simulated two scenarios corresponding to the presence or absence of antibody cross- reaction between species. In the simulations all host specific responses were maintained, transferring all cross-infecting pathogens’ specific responses, for example the effect of ACT on the interaction between antigen presenting cells and naïve T cells was maintained during the *B. bronchiseptica* after *B. pertussis* cross-infection .

In the cross-reacting scenario *B. pertussis* was cleared on the 18^th^ time-step and *B. bronchiseptica* was cleared on the 21^st^ time-step during infection of diseased hosts (in phase II). For secondary infection of convalescent hosts (phase III) both species of bacteria were cleared in 6 time-steps. Thus cross-infection in the presence of cross-reacting antibodies is predicted to have a similar timecourse as a secondary infection by the same species, consistent with preliminary experimental data (Harvill lab, unpublished).

The absence of cross-reactivity was simulated by switching off the nodes corresponding to dendritic cells, T0 cells, Th2 cells, B cells and antibodies (complement fixing or otherwise) from the secondary initial state, reflecting that the new antigens need to be recognized to induce B cell clonal expansion. *B. pertussis* delivered in phase II or III of *B. bronchiseptica* were cleared on the 20^th^ time-step (see Table S5), very close to the clearance timescale in naïve mice. Although Th1 related cytokines were active, the absence of antibodies that recognize new antigen delayed clearance. Secondary *B. bronchiseptica* given in phase II of *B. pertussis* infection were cleared earlier than those given in phase III (in the 21^st^ step compared to the 26^th^). Bacteria given in phase III in essence followed the timecourse of *B. bronchiseptica* in naïve mice.

In the *B. pertussis* cross infection (following *B. bronchiseptica*), phagocytosis, pro-inflammatory cytokines and recruited PMNs were active for more time-steps as compared to a *B. pertussis* (primary) infection. This can be attributed to the fact that the more pathogenic responses elicited by *B. bronchiseptica* lead to a continued activation of those responses at the time of secondary infection*.* On the other hand *B. bronchiseptica* cross-infection shows a reduced recruitment of PMNs as compared to a *B. bronchiseptica* (primary) infection. Delayed activation of some nodes, for example of the Ag/Ab complex, was observed in the model with no cross-reactive antibodies. Nevertheless the earlier clearance of most of the secondary challenges indicates that the cytokines and other nodes activated in the primary infection assist the clearance of the secondary infection.
