# Supplementary material for: Modeling Systems-Level Regulation of Host Immune Responses
Source: PLoS Comput Biol. 2007 Jun 8;3(6):e109. doi: 10.1371/journal.pcbi.0030109 (PMC1892604; doi:10.1371/journal.pcbi.0030109)
Supplement: Supplementary file 6 [file pcbi.0030109.st001.doc]

**Table S1. Synthesis of experimental information about regulatory interactions between host immune components and bacterial virulence factors.**

Each entry in the database consists of a source node (Node A), a target node or target process (Node/Process B), their interaction, and references. In the column “Node B” → means activation of the target node (given after the arrow) by the source node (indicated before the arrow). We classify interactions into two categories: “promotes” (that can represent synthesis, transformation, upregulation or activation) and “inhibits” (that can represent catalyzed decay, downregulation or negative interference). “A promotes B” is usually inferred if in the absence of A, B cannot be observed; “A inhibits B” is inferred if in the absence of A, B is upregulated. The reference list gives representative references or indicates that the interaction is part of the core immunological knowledge; in some cases the phenomenon was described in multiple reports, and additional literature references are given in the main text.

| **Node A** | **Node B** | **Interaction** | **Reference** |
| --- | --- | --- | --- |
| Bacteria | Epithelial cells | Promotes | Basic Immunology |
| Bacteria | Complement | Promotes | Basic Immunology |
| Bacteria | Dendritic cells (DC) | Promotes | [1] |
| Epithelial cells | Pro-inflammatory cytokines (PIC) | Promotes | [2] |
| PIC | Recruited PMNs | Promotes | [2] |
| PIC | Macrophages | Promotes | [2] |
| PIC | DC | Promotes | Basic Immunology |
| Recruited PMNs | Activated phagocytic cells (ActPhagCells) | Promotes | Basic Immunology |
| Type III secretion system (TTSS) | Necrosis of Recruited PMNs | Promotes | [3] |
| ActPhagCells | PIC | Promotes | Basic Immunology |
| ActPhagCells | Phagocytosis | Promotes | [4] |
| Macrophages | ActPhagCells | Promotes | Basic Immunology |
| Complement | ActPhagCells | Promotes | Basic Immunology |
| Complement fixing Ab | Antigen-Antibody Complex (AgAbComplex) | Promotes | Basic Immunology |
| Other Ab | AgAbComplex | Promotes | Basic Immunology |
| AgAbComplex | ActPhagCells | Promotes | Basic Immunology |
| AgAbComplex | Complement | Promotes | Basic Immunology |
| Complement fixing Ab | AgAbComplex🡪Complement | Promotes | Basic Immunology |
| B cells | Complement fixing Ab | Promotes | Basic Immunology |
| B cells | Other Ab | Promotes | Basic Immunology |
| Th2 cells | B cells | Promotes | Basic Immunology |
| Th2 cells | Th2 related cytokines (Th2RC) | Promotes | Basic Immunology |
| Th1 cells | Th1 related cytokines (Th1RC) | Promotes | Basic Immunology |
| Th1RC | Macrophages | Promotes | Basic Immunology |
| Th1RC | T0 cells 🡪Th1 cells | Promotes | [5] |
| Th1RC | DC | Promotes | [5] |
| Th1RC | Th2RC | Inhibits | [5] |
| Th2RC | T0 cells🡪 Th2 cells | Promotes | [5] |
| Th2RC | DC | Promotes | [5] |
| Th2RC | Th1RC | Inhibits | [5,6] |
| Th2RC | PIC | Inhibits | Basic Immunology |
| DC | Th2RC | Promotes | [5] |
| DC | Th1RC | Promotes | [5] |
| DC | T0 cells | Promotes | [1] |
| FHA/ACT | DC🡪Th2RC | Promotes | [7] |
| TTSS | Th1RC | Inhibits | [6] |
| PTX | Recruited PMNs | Inhibits | [8,9] |
| O Antigen (OAg) | Bacteria🡪Complement | Inhibits | [10] |
| Phagocytosis | Bacteria | Inhibits | Basic Immunology, [4] |

**References**

1. Reis e Sousa C (2004) Activation of dendritic cells: translating innate into adaptive immunity. Curr Opin Immunol 16: 21-25.

2. Mann PB, Elder KD, Kennett MJ, Harvill ET (2004) Toll-like receptor 4-dependent early elicited tumor necrosis factor alpha expression is critical for innate host defense against Bordetella bronchiseptica. Infect Immun 72: 6650-6658.

3. Yuk MH, Harvill ET, Cotter PA, Miller JF (2000) Modulation of host immune responses, induction of apoptosis and inhibition of NF-kappaB activation by the Bordetella type III secretion system. Mol Microbiol 35: 991-1004.

4. Pishko EJ, Kirimanjeswara GS, Pilione MR, Gopinathan L, Kennett MJ, et al. (2004) Antibody-mediated bacterial clearance from the lower respiratory tract of mice requires complement component C3. Eur J Immunol 34: 184-193.

5. O'Garra A, Arai N (2000) The molecular basis of T helper 1 and T helper 2 cell differentiation. Trends Cell Biol 10: 542-550.

6. Pilione MR, Harvill ET (2006) The Bordetella bronchiseptica type III secretion system inhibits gamma interferon production that is required for efficient antibody-mediated bacterial clearance. Infect Immun 74: 1043-1049.

7. Boyd AP, Ross PJ, Conroy H, Mahon N, Lavelle EC, et al. (2005) Bordetella pertussis Adenylate Cyclase Toxin Modulates Innate and Adaptive Immune Responses: Distinct Roles for Acylation and Enzymatic Activity in Immunomodulation and Cell Death. J Immunol 175: 730-738.

8. Carbonetti NH, Artamonova GV, Mays RM, Worthington ZE (2003) Pertussis toxin plays an early role in respiratory tract colonization by Bordetella pertussis. Infect Immun 71: 6358-6366.

9. Kirimanjeswara GS, Agosto LM, Kennett MJ, Bjornstad ON, Harvill ET (2005) Pertussis toxin inhibits neutrophil recruitment to delay antibody-mediated clearance of Bordetella pertussis. J Clin Invest 115: 3594-3601.

10. Burns VC, Pishko EJ, Preston A, Maskell DJ, Harvill ET (2003) Role of Bordetella O antigen in respiratory tract infection. Infect Immun 71: 86-94.
