# Supplementary material for: Modeling Systems-Level Regulation of Host Immune Responses
Source: PLoS Comput Biol. 2007 Jun 8;3(6):e109. doi: 10.1371/journal.pcbi.0030109 (PMC1892604; doi:10.1371/journal.pcbi.0030109)
Supplement: Supplementary file 7 [file pcbi.0030109.st002.doc]

**Table S2. Temporal information known from experimental observations in *B. bronchiseptica* (Bb) and *B. pertussis* (Bp).**

| **Node** | **Species** | **Peak/first activity** | **Description/ Significance** | **Reference** |
| --- | --- | --- | --- | --- |
| PIC | Bb/Bp | 2-24 hours | Pro-inflammatory cytokines respond to the pathogen invasion in the first 24 hours. | [1] |
| Recruited PMNs | Bb | 12 hours | First peak of recruited PMNs is at 12 hours. This is a nonspecific innate immune response. | [2] |
| Necrosis of recruited PMNs | Bb | 3-4 days | In *B. bronchiseptica* the TTSS induces necrosis of PMNs between the 3^rd^ and 4^th^ day. This indicates a delay in the action of TTSS after bacterial invasion. | [3,4] |
| Start of phagocytosis | Bb | 12 hours | Non-specific phagocytosis starts12 hours after infection. | [2] |
| Dendritic cells | Bp | 4-7 days  15-21 days | In *B. pertussis* two peaks of dendritic cells are observed, supporting the differentiation of distinct subsets of T helper cells from T0 cells. | [1] |
| T0 cells | Bb | 3-7 days | In *B. bronchiseptica* T0 cells are first observed during the first week of infection. | Our unpublished data, [5] |
| Th2 cells | Bb | 8 days | Th2 cells are observed after the first peak of dendritic cells. | [5] |
| IL10 (a Th2RC) | Bb/Bp | 3-7 days | First peak of IL10 is observed during the initial interaction between dendritic cells and T0 cells. | [1] |
| Antibodies | Bb | 8-14 days | Antibodies are first observed on the 8^th^ day, that is, after the activation of Th2 cells. | [6] |
| AgAb Complex | Bb | 9-21 days | Antigen-antibody complexes are observed between the 9^th^ and 21^st^ day. | [6] |
| IFN-γ (a Th1RC) | Bb | 49-70 days | IFN-γ is produced later, around day 49, in *B. bronchiseptica*. | [5] |
| NK cells | Bp | Day 15^th^ onwards | Natural killer cells are seen after day 15 during *B. pertussis* infection. They are also a source of IFN-γ. | [1] |

**References**

1. Byrne P, McGuirk P, Todryk S, Mills KH (2004) Depletion of NK cells results in disseminating lethal infection with Bordetella pertussis associated with a reduction of antigen-specific Th1 and enhancement of Th2, but not Tr1 cells. Eur J Immunol 34: 2579-2588.

2. Mann PB, Elder KD, Kennett MJ, Harvill ET (2004) Toll-like receptor 4-dependent early elicited tumor necrosis factor alpha expression is critical for innate host defense against Bordetella bronchiseptica. Infect Immun 72: 6650-6658.

3. Stockbauer KE, Foreman-Wykert AK, Miller JF (2003) Bordetella type III secretion induces caspase 1-independent necrosis. Cell Microbiol 5: 123-132.

4. Yuk MH, Harvill ET, Cotter PA, Miller JF (2000) Modulation of host immune responses, induction of apoptosis and inhibition of NF-kappaB activation by the Bordetella type III secretion system. Mol Microbiol 35: 991-1004.

5. Skinner JA, Pilione MR, Shen H, Harvill ET, Yuk MH (2005) Bordetella type III secretion modulates dendritic cell migration resulting in immunosuppression and bacterial persistence. J Immunol 175: 4647-4652.

6. Kirimanjeswara GS, Mann PB, Harvill ET (2003) Role of antibodies in immunity to Bordetella infections. Infect Immun 71: 1719-1724.
