# Supplementary material for: Modeling Systems-Level Regulation of Host Immune Responses
Source: PLoS Comput Biol. 2007 Jun 8;3(6):e109. doi: 10.1371/journal.pcbi.0030109 (PMC1892604; doi:10.1371/journal.pcbi.0030109)
Supplement: Supplementary file 8 [file pcbi.0030109.st003.doc]

**Table S3. Summary of experimental data on the effect of bacteria/mouse knockout mutations on bacterial clearance. This information was used to test the model’s outcomes in the corresponding simulated knockouts. For comparison the timing of the wild type infections is also given.**

| **Mutant** | **Species** | **Timing of bacterial clearance (days post inoculation)** | **Reference** |
| --- | --- | --- | --- |
| Wild type | Bb  Bp | 70  50 | [1] |
| Deletion of TTSS | Bb | 27 | [2] |
| Deletion of PTX | Bp | 50 | [3] |
| B cell deletion | Bb/Bp | Bacterial persistence | [1] |
| Absence of adaptive immunity | Bb/Bp | Bacterial persistence | [4] |
| Antibody treatment | Bb  Bp | 3  50 | [1] |

**References**

1. Kirimanjeswara GS, Mann PB, Harvill ET (2003) Role of antibodies in immunity to Bordetella infections. Infect Immun 71: 1719-1724.

2. Pilione MR, Harvill ET (2006) The Bordetella bronchiseptica type III secretion system inhibits gamma interferon production that is required for efficient antibody-mediated bacterial clearance. Infect Immun 74: 1043-1049.

3. Kirimanjeswara GS, Agosto LM, Kennett MJ, Bjornstad ON, Harvill ET (2005) Pertussis toxin inhibits neutrophil recruitment to delay antibody-mediated clearance of Bordetella pertussis. J Clin Invest 115: 3594-3601.

4. Harvill ET, Cotter PA, Miller JF (1999) Pregenomic comparative analysis between bordetella bronchiseptica RB50 and Bordetella pertussis tohama I in murine models of respiratory tract infection. Infect Immun 67: 6109-6118.
