# Supplementary material for: Modeling Systems-Level Regulation of Host Immune Responses
Source: PLoS Comput Biol. 2007 Jun 8;3(6):e109. doi: 10.1371/journal.pcbi.0030109 (PMC1892604; doi:10.1371/journal.pcbi.0030109)
Supplement: Supplementary file 9 [file pcbi.0030109.st004.doc]

**Table S4. Nodes active in (a portion of) each phase of the infection with *B. bronchiseptica* (Bb) and *B. pertussis* (Bp).**

| **Phase of the infection** | **Node** | **Duration of activity (timesteps)** | |
| --- | --- | --- | --- |
|  |  | **Bb** | **Bp** |
| I | Bacteria | 5 | 3 |
| I | Epithelial cells | 4-5 | 1-3 |
| I | PIC | 4-5 | 2-3 |
| I | Macrophages | 4-5 | 1-2 |
| I | Dendritic cells | 4-5 | 3 |
| I | O Antigen | 5 | - |
| I | TTSS | 5 | - |
| I | PTX | - | 3 |
| I | FHA/ACT | - | 3 |
| II | Bacteria | 15 | 13 |
| II | PTX | - | 1 |
| II | Complement | 12 | 13 |
| II | Dendritic cells | 15 | 13 |
| II | T0 cells | 15 | 13 |
| II | Th2 cells | 15 | 13 |
| II | Th2 related cytokines | 15 | 13 |
| II | Dead PMNs | 1 | - |
| II | TTSS | 15 | - |
| II | B cells | 14 | 13 |
| II | Complement fixing antibodies | 12 | 12 |
| II | Other antibodies | 12 | 12 |
| III | Bacteria | 5 | 5 |
| III | PIC | 4 | 5 |
| III | Complement | 5 | 5 |
| III | Recruited PMNs | 3 | 4-5 |
| III | Macrophages | 4-5 | 4-5 |
| III | Activated phagocytic cells | 4 | 4 |
| III | Dendritic cells | 5 | 5 |
| III | T0 cells | 5 | 5 |
| III | Th1 cells | 3-5 | 3-5 |
| III | Th1 related cytokines | 4-5 | 4-5 |
| III | Complement fixing antibodies | 5 | 5 |
| III | Other antibodies | 5 | 5 |
| III | Phagocytosis | 4 | 4 |
