# Supplementary material for: Modeling Systems-Level Regulation of Host Immune Responses
Source: PLoS Comput Biol. 2007 Jun 8;3(6):e109. doi: 10.1371/journal.pcbi.0030109 (PMC1892604; doi:10.1371/journal.pcbi.0030109)
Supplement: Supplementary file 10 [file pcbi.0030109.st005.doc]

**Table S5. *B. bronchiseptica* and *B. pertussis* clearance time-steps during simulated cross-infections.**

| **Primary infection**  **(PI)** | **Secondary infection**  **(SI)** | **Phase of PI during secondary invasion** | **Antibody cross reactivity** | **Time-step of bacterial clearance** |
| --- | --- | --- | --- | --- |
| Bb | Bp | Phase II  Phase III | yes | 18  6 |
| Bb | Bp | Phase II  Phase III | no | 20  20 |
| Bp | Bb | Phase II  Phase III | yes | 21  6 |
| Bp | Bb | Phase II  Phase III | no | 21  26 |
